# PTEN phosphatase inhibits metastasis by negatively regulating the Entpd5/IGF1R pathway through ATF6

**DOI:** 10.1016/j.isci.2023.106070

**Published:** 2023-01-26

**Authors:** Yanlin Yu, Meng Dai, Liping Huang, Weiping Chen, Ellen Yu, Arnulfo Mendoza, Helen Michael, Chand Khanna, Marcus Bosenberg, Martin McMahon, Glenn Merlino

**Affiliations:** 1 Laboratory of Cancer Biology and Genetics, National Cancer Institute, National Institutes of Health, Bethesda, MD 20892, USA; 2 Southern Medical University, Guangzhou, People’s Republic of China; 3 NIDDK, National Institutes of Health, Bethesda, MD 20892, USA; 4 Pediatric Oncology Branch, National Cancer Institute, National Institutes of Health, Bethesda, MD 20892, USA; 5 Yale University, New Haven, CT 06520, USA; 6 Huntsman Cancer Institute, University of Utah, Salt Lake City, UT 84112, USA

**Keywords:** Cell biology, Functional aspects of cell biology, Cancer

## Abstract

*PTEN* encodes a tumor suppressor with lipid and protein phosphatase activities whose dysfunction has been implicated in melanomagenesis; less is known about how its phosphatases regulate melanoma metastasis. We demonstrate that PTEN expression negatively correlates with metastatic progression in human melanoma samples and a PTEN-deficient mouse melanoma model. Wildtype PTEN expression inhibited melanoma cell invasiveness and metastasis in a dose-dependent manner, behaviors that specifically required PTEN protein phosphatase activity. PTEN phosphatase activity regulated metastasis through Entpd5. Entpd5 knockdown reduced metastasis and IGF1R levels while promoting ER stress. In contrast, Entpd5 overexpression promoted metastasis and enhanced IGF1R levels while reducing ER stress. Moreover, Entpd5 expression was regulated by the ER stress sensor ATF6. Altogether, our data indicate that PTEN phosphatase activity inhibits metastasis by negatively regulating the Entpd5/IGF1R pathway through ATF6, thereby identifying novel candidate therapeutic targets for the treatment of PTEN mutant melanoma.

## Highlights

• PTEN expression negatively correlates with metastatic progression in melanoma
• PTEN functions as a haploinsufficiency metastasis suppressor
• PTEN phosphatase is required for blocking metastasis independent of lipid phosphatase
• PTEN regulated metastasis by inhibiting Entpd5/IGF1R/ATF6 pathways

## Graphical abstract

**Figure:**
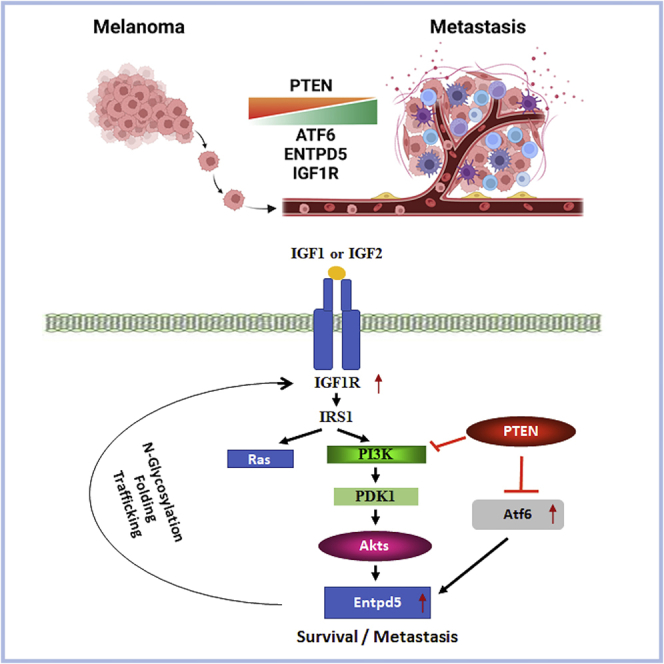

## Introduction

The functional disruption of phosphatase and tensin homolog deleted on chromosome ten (PTEN) by genetic mutation or epigenetic silencing contributes to the development and progression of most cancers^1^^,^^2^^,^^3^ and is often associated with high grade and metastatic potential as well as poor patient prognosis.^3^^,^^4^^,^^5^^,^^6^^,^^7^ Inactivation of PTEN is caused by various mechanisms including genetic loss, point mutation, epigenetic regulation and posttranslational modifications. Most alterations result in the loss or reduction of PTEN protein. In mice, total PTEN loss leads to embryonic lethality, and even a small reduction in PTEN levels enhances cancer incidence.^7^^,^^8^^,^^9^^,^^10^^,^^11^ Conversely, the systemic overexpression of PTEN in genetically engineered mice (GEM) augments its tumor-suppressive function and protects tumorigenesis.^12^ Thus, the precise level of PTEN expression is a critical factor for tumor suppressor function, and the reduction of PTEN activity is a driving mechanism for tumor progression.

The pathologic features associated with PTEN deletion in mice are phenocopied in a wide range of human tumors with loss of heterozygosity (LOH) mutation of PTEN. In melanoma, the PI3K/PTEN/Akt pathway has been identified as one of the key molecular lesions governing melanomagenesis.^13^^,^^14^^,^^15^^,^^16^ Loss of PTEN function has been reported to be responsible for many of the phenotypic features of melanoma and affects the development of 30–50% of human melanomas.^3^^,^^14^^,^^15^ Recent studies indicate that absent or reduced PTEN protein levels have been found in 62%–65% of metastatic melanoma.^16^^,^^17^^,^^18^ Moreover, lower PTEN dosage is correlated with tumor progression. For instance, patients with *PTEN* gene methylation, an epigenetic modification associated with a decrease in gene transcription level, have a significantly higher risk of dying compared with patients without such methylation.^19^ Low PTEN expression is also associated with melanoma ulceration, which is a characteristic of aggressive tumors.^20^ In contrast, PTEN inactivation is rare in benign nevi,^21^^,^^22^ implying that PTEN may play an important role in the later stages of melanoma progression.^1^^,^^2^^,^^16^

*PTEN* encodes a tumor suppressor with both lipid and protein phosphatase activities.^2^^,^^23^*In vitro*, recombinant PTEN has been shown to dephosphorylate protein substrates on serine, threonine and tyrosine residues.^2^^,^^23^ Moreover, PTEN protein phosphatase has been proposed to dephosphorylate focal adhesion kinase (FAK),^24^ c-Src,^25^ and PTEN itself,^26^ thereby inhibiting cell adhesion and migration and has been implicated in maintaining genomic integrity through dephosphorylation of H2AX and the recruitment of Rad51 and 53bp1 to sites of DNA damage.^27^ The PTEN lipid phosphatase is known to dephosphorylate the plasma member lipid phosphatidyl-inositol-3, 4, 5-trisphosphate (PIP3). This enables PTEN to effectively antagonize the PI3K pathway, resulting in the inactivation of many downstream protein kinases, such as PDK1 and AKT, thereby inhibiting cell proliferation and promoting apoptosis.^28^^,^^29^

Much has been learned about PTEN through analysis of commonly occurring missense mutations, including those found in exon 5 encoding the phosphatase domain, which can alter PTEN phosphatase activity.^30^ Patients with Cowden disease (CD), who harbor missense PTEN mutations in exon 5 and are cancer-prone, develop a higher number of lesions compared to patients with truncating mutations causing complete loss of PTEN function.^31^ Importantly, one CD-derived mutation in PTEN (G129E),^32^ which loses lipid but not protein phosphatase activity, cannot induce either G1 arrest or apoptosis but nonetheless retains the ability to inhibit cell spreading and motility. Another CD-derived PTEN mutant (C124S), which lacks both lipid and protein phosphatase activity, is unable to block cell spreading or migration,^24^^,^^26^ suggesting that PTEN protein phosphatase activity may play an important role in tumor cell migration.

Of interest, PTEN missense mutations in the phosphatase core also occur more often in metastatic melanoma compared to matched primary lesions from the same patient.^33^ Birck et al. reported that allelic loss of PTEN was found in 38% of primary melanoma and 58% of melanoma metastasis, whereas missense mutations occurred in 7% of metastatic melanomas but in none of the primary tumors studied.^18^ Although a few studies have shown that PTEN reconstitution or overexpression in PTEN-deficient tumor cells inhibits tumor cell migration,^16^^,^^24^^,^^26^ the mechanism by which the level of PTEN regulates metastasis, or which of the PTEN specific phosphatase activities are involved, remains to be clarified. Moreover, little *in vivo* experimental evidence directly demonstrates a role for the PTEN phosphatases in tumor metastasis.

We here use a variety of approaches to demonstrate that PTEN expression can regulate metastatic behavior. Forced expression of wildtype PTEN reduces the metastatic potential of melanoma cells in a dose-dependent manner, whereas expression of a PTEN phosphatase mutant promotes metastatic melanoma behavior in a dominant-negative manner. Notably, we demonstrate that PTEN phosphatase negatively regulates the protein N-glycosylation and folding promoter Entpd5 and the Entpd5/IGF1R pathway through Atf6 transcriptional regulation, identifying an important new mechanism by which PTEN inhibits melanoma metastasis.

## Results

### PTEN deficiency induces melanoma progression in genetically engineered mice

**Figure 1:**
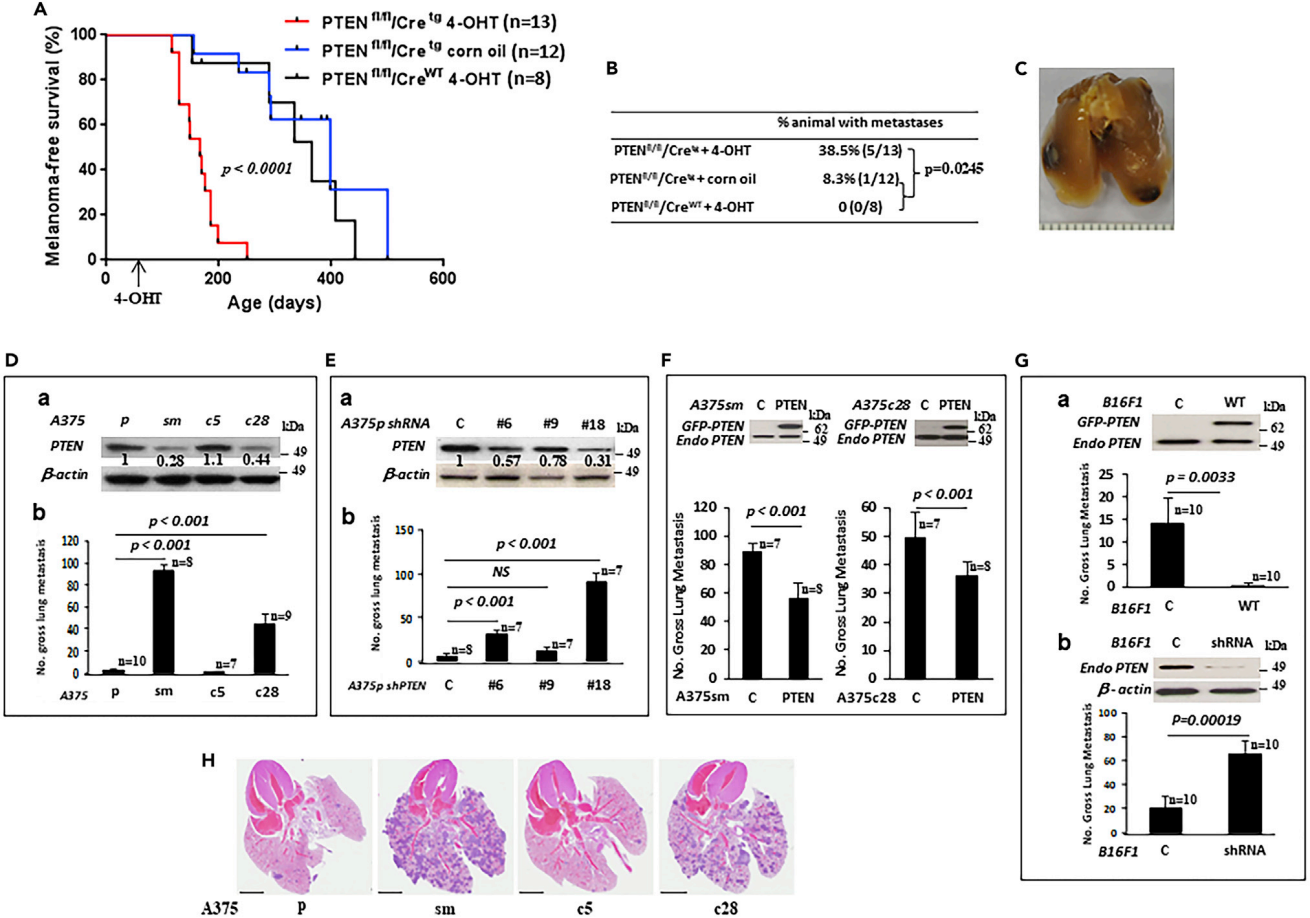
PTEN expression is correlated to metastatic behavior in melanoma (A) PTEN loss led to markedly reduced survival in the UV-irradiated HGF transgenic mouse model. HGF^Tg/+^/PTEN^fl/fl^/Cre^Tg/+^ and HGF^Tg/+^/PTEN^fl/fl^/Cre^+/+^ mice were developed by crossing the HGF^Tg/+^, PTEN^fl/fl^ and Tyr:CreER^Tg/Tg^ GEM models. Melanomas were initiated by a single dose of UV radiation at 3.5 days of age. The depletion of PTEN was achieved by administration of 4-hydroxytamoxifen (4-OHT) at 8 weeks of age. Kaplan-Meier survival analysis of 4-OHT-treated HGF^Tg/+^/PTEN^fl/fl^/Cre^Tg/+^ (n = 13), HGF^Tg/+^/PTEN^fl/fl^/Cre^+/+^ (n = 12), and corn oil treated-HGF^Tg/+^/PTEN^fl/fl^/Cre^Tg/+^ (n = 8) mice. Log-rank tests of survival plots of the data indicated a statistically significant difference between the following survival curves: HGF^Tg/+^/PTEN^fl/fl^/Cre^Tg/+^ with 4-OHT vs. HGF^Tg/+^/PTEN^fl/fl^/Cre^Tg/+^ with corn oil (p<0.0001) and HGF^Tg/+^/PTEN^fl/fl^/Cre^Tg/+^ with 4-OHT vs. HGF^Tg/+^/PTEN^fl/fl^/Cre^+/+^ with 4-OHT (p<0.0001). There was no statistically significant difference between HGF^Tg/+^/PTEN^fl/fl^/Cre^Tg/+^ with corn oil vs. HGF^Tg/+^/PTEN^fl/fl^/Cre^+/+^ with 4-OHT. (B) Incidence of metastasis in mice. PTEN-deficient UV-irradiated HGF^tg/+^ mice suffered a significantly higher risk of metastasis than PTEN-WT UV-irradiated HGF^tg/+^ mice, Fisher exact test p = 0.0248. (C) Representative lung image with metastatic lesion from HGF^Tg/+^/PTEN^fl/fl^/Cre^Tg/+^ mouse with corn oil. (D) PTEN expression and gross pulmonary metastases from a panel of well-established human melanoma A375p (p), A375sm (sm), A375c5 (c5) and A375c28 (c28) cell lines. (a) PTEN protein level by western blot; the quantitated data of the fold changes normalized with β-actin; (b) gross pulmonary metastases by tail-vein injection. In comparison with p cells, sm and c28 cells had significantly increased pulmonary metastatic potential (p <0.001). (E) Knockdown of endogenous PTEN by shRNA enhanced metastatic potential in A375p cells. (a) three stable cell clones expressing reduced PTEN to different degrees by 43%, 22% and 69%, respectively; (b) gross pulmonary metastases. Compared to control c, three clones showed variously increased pulmonary metastasis; clone #6 and #18 had a statistically significant increase in metastasis (p<0.001). (F) Overexpression of PTEN in human melanoma A375sm and A375c28 cells significantly inhibited pulmonary metastases (p < 0.001). C, empty vector; PTEN, overexpression of PTEN. (G) Overexpression of PTEN in mouse melanoma B16F1 cells significantly inhibited pulmonary metastases (p = 0.0033) (a), whereas knockdown of endogenous PTEN enhanced pulmonary metastases significantly (p = 0.00019) (b). C, empty vector; WT, overexpression of PTEN; shRNA, knockdown of endogenous PTEN by shRNA for PTEN. (H) Representative H&E stained lung sections are shown for the experimental metastases from a panel of well-established human melanoma A375p (p), A375sm (sm), A375c5 (c5) and A375c28 (c28) cell lines (D) (scale bar = 5 mM). Data represented as mean ± SEM for all columns. The p value is shown by an unpaired t-test (two-tailed).

To validate the importance of PTEN in melanoma progression *in situ*, we established conditional PTEN loss in the context of the hepatocyte growth factor/scatter factor (HGF) autochthonous melanoma GEM model, in which a single initiating neonatal dose of UV radiation causes premalignant melanocytic lesions (nevi) by 2–3 months, some of which then progress over the next 6 to 12 months to invasive, metastatic melanoma.^34^ HGF^Tg^, PTEN^fl^ and Tyr:Cre^Tg^ alleles^35^ were combined to establish HGF^Tg/+^/PTEN^fl/fl^/Tyr:Cre^Tg/+^ GEMs, which were subjected to standard melanoma-initiating neonatal UV-radiation. At 8 weeks of age, after early-stage melanocytic lesions were apparent, HGF^Tg/+^/PTEN^fl/fl^/Tyr:Cre^Tg/+^ mice were treated with 4-OHT (tamoxifen) to induce PTEN loss, and subsequently exhibited significantly shorter longevity in comparison to HGF^Tg/+^/PTEN^fl/fl^/Tyr:Cre^Tg/+^ mice treated with corn oil or HGF^Tg/+^/PTEN^fl/fl^/Tyr:Cre^+/+^ mice treated with 4-OHT (Figure 1A). Advanced malignant melanoma lesions in PTEN-deficient UV-irradiated HGF^tg/+^ mice were often associated with metastasis as well (compared with PTEN-WT UV-irradiated HGF^tg/+^ mice, Fisher exact test p = 0.0248; Figures 1B and 1C), implying that PTEN deficiency may play an important role in melanoma progression. Moreover, we have evaluated the Skin Cutaneous Melanoma (SKCM-TCGA) dataset and found that the patients with PTEN downregulation correlate with worse survival in SKCM-TCGA (Figure S1). Also, the metastatic melanoma patients with PTEN mutations (deletion and mutation) have significantly worse survival in SKCM-TCGA metastatic melanoma (MSKCC, JCO Precis Oncol 2017)^36^ dataset (Figure S2). Moreover, we found that PTEN methylation negatively correlates with survival probability in SKCM-TCGA data (Figure S3A). We conclude that loss of PTEN function could play a significant role in melanoma progression as well as metastasis.

### PTEN expression suppresses metastasis in a dose-dependent manner

GEM studies have demonstrated that PTEN can function as a haploinsufficiency tumor suppressor.^8^^,^^9^^,^^10^ We therefore examined the consequences of altering PTEN expression levels on metastasis, focusing on a panel of well-established human melanoma cell lines (A375p, A375sm, A375c5, A375c28), derived from the same human melanoma tumor with different metastatic potential. The parent cell line, A375p, is poorly metastatic, whereas the other three cell lines, A375sm, A375c5 and A375c28, were developed from A375p through *in vivo* selection. We first determined PTEN expression by western blot in these cells (Figure 1D-a). PTEN protein levels were similar in A375p and A375c5 cells but decreased by 72% and 56% in A375sm and A375c28 cells, respectively, relative to A375p (Figure 1D-a). We noted that the cell lines with lower levels of PTEN protein demonstrated a higher degree of pulmonary metastasis *in vivo*; in contrast, those with higher levels of PTEN exhibited less pulmonary metastasis (Figures 1D-b and 1H). These results supported the notion that PTEN expression levels may be related to metastatic behavior. To confirm this, we knocked down endogenous PTEN in A375p cells using shRNA and generated three stable cell clones expressing PTEN at levels that were reduced by 43%, 22% and 69% (Figure 1E-a). Notably, reductions in PTEN levels led to corresponding enhancements in metastatic potential (Figures 1E-b and S4A). In contrast, the elevation of PTEN in both human melanoma cell lines A375sm and A75c28 with a lower level of PTEN caused in reduction of metastasis (Figures 1F, S4B and S4C). Results using mouse melanoma cells yielded similar results: overexpression of PTEN in mouse melanoma B16F1 cells inhibited pulmonary metastasis (Figures 1G-a and S4D), whereas knockdown of endogenous PTEN by shRNA enhanced pulmonary metastasis (Figures 1G-b and S4E). These data suggest that metastatic behavior correlates with the expression level of PTEN and that PTEN can influence metastatic potential in a dose-dependent manner.

### PTEN phosphatase activity is required for suppressing metastasis

**Figure 2:**
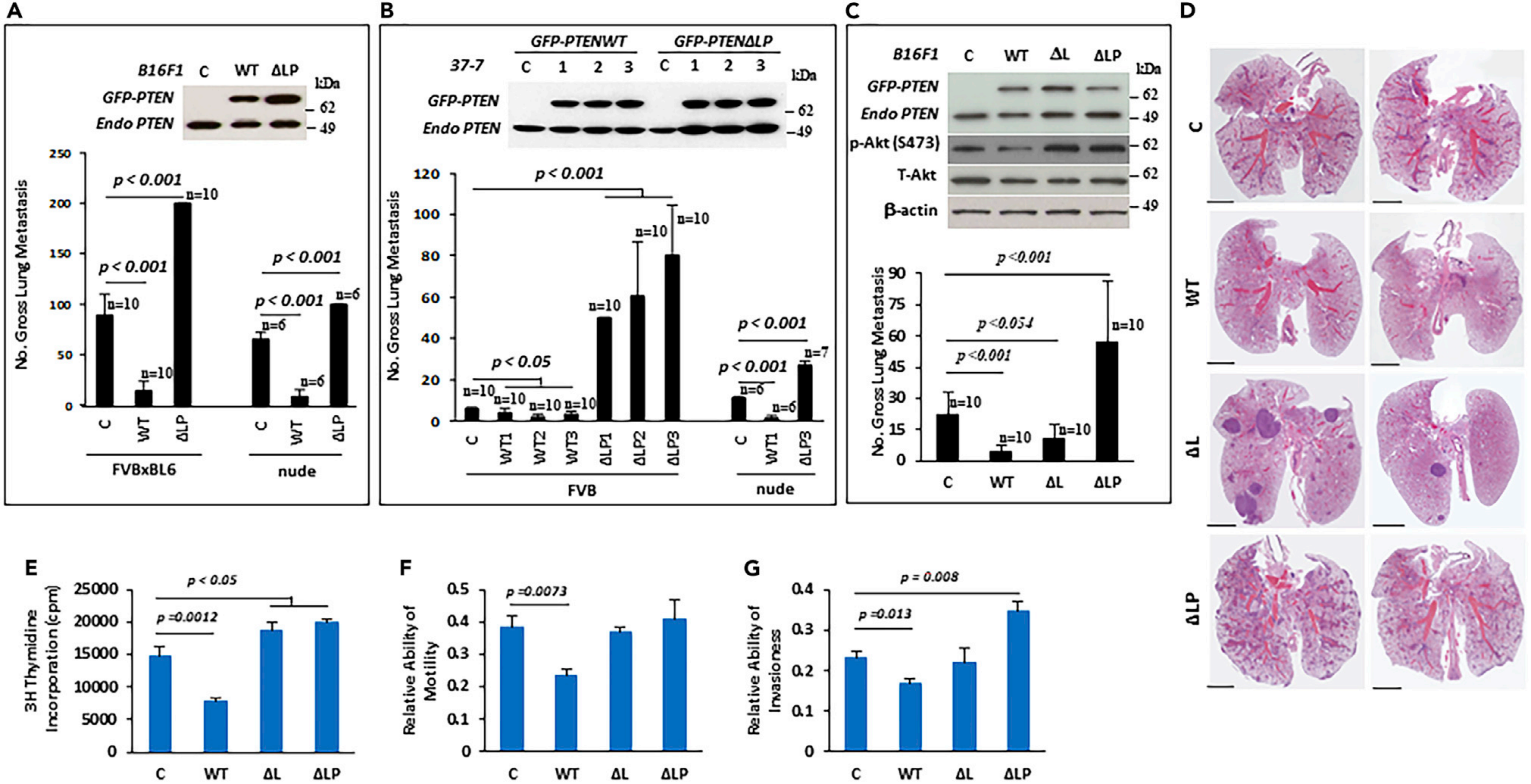
PTEN regulates metastasis through its phosphatase activity independent of lipid phosphatase activity (A and B) PTEN phosphatase dead mutant enhanced metastasis. Western blot analysis of lysates from B16F1 (A, top panel) or 37-7 cell (B, top panel) transfectants harboring empty vector (c), PTEN wildtype (WT or GFP-PTEN) and PTEN phosphatase dead mutant (ΔLP or GFP-PTEN ΔLP) and gross pulmonary metastases in different hosts (FVB host with 1x10e6 cells, nude host with 1x10e5 cells) *in vivo* (bottom panel of A or B). c, empty vector control; 1, 2 and 3 in 37-7 cells as three different clones. (C) PTEN lipid phosphatase mutant was not required for suppression of metastasis. Enforced expression of various PTEN mutants in B16F1 cells was represented by western blot (top panel) and gross pulmonary metastases from B16F1 cell transfectants (bottom panel). C, empty vector; WT, PTEN wildtype; ΔL, PTEN lipid phosphatase deficient; ΔLP, PTEN phosphatase dead mutant. (D) Representative histopathology (H&E staining) of lung sections with metastases from mice bearing B16F1 cells with empty vector (c), PTEN WT (WT), PTEN lipid phosphatase deficient (ΔL), PTEN phosphatases dead mutant (ΔLP) (scale bar = 5 mM). (E–G) Phenotypic effects of various PTEN mutants in B16F1 *in vitro*. The proliferation rates (E) of cells were assessed by measuring the incorporation of [^3^H] thymidine. Motility (F) and invasiveness (G) were determined using Transwell culture plates for 12 or 48 h, respectively. C, vector alone; WT, PTEN wildtype; ΔL, PTEN lipid phosphatase deficient; ΔLP, PTEN phosphatase dead mutant. B16F1 cells transfected with PTEN wildtype (WT) demonstrated significant inhibition in growth (p = 0.0012), motility (p = 0.0073) and invasiveness (p = 0.013) *in vitro* compared with empty vector control (c). The cells expressing PTEN phosphatase dead mutant (ΔLP) or PTEN lipid phosphatase deficient (ΔL) showed a significant increase in cell growth (p<0.05), but no significant difference in cell motility. However, the cells with PTEN phosphatase dead mutant (ΔLP) showed an increase in cell invasiveness (p = 0.008). Data is representative of three independent experiments. Graphs show the mean ± SEM. The p value is shown by an unpaired t-test (two-tailed).

To determine whether PTEN phosphatase activity is required for suppressing melanoma metastasis, wildtype PTEN (PTEN WT) or the phosphatase dead mutant C124S (PTEN ΔLP) were introduced into B16F1 and 37-7^37^ mouse melanoma cell lines. Stably transfected cells were selected for testing metastatic potential in syngeneic and nude mice *in vivo*. As shown in Figures 2A and 2B, expression of PTEN WT significantly inhibited pulmonary metastasis in both B16F1 (Figures 2A, S5A and S5B) and 37-7 (Figures 2B, S5C and S5D) cell lines in two different hosts. In contrast, PTEN ΔLP stimulated pulmonary metastasis in both cell lines. These data further confirmed the role of PTEN in negatively regulating metastasis and indicated that the PTEN ΔLP had a dominant-negative effect^38^ on PTEN while promoting melanoma metastasis. Moreover, PTEN phosphatase activity was required for suppressing metastasis in melanoma.

### PTEN phosphatase-mediated metastatic suppression does not require its lipid phosphatase activity

The phosphatase activity of PTEN includes the ability to dephosphorylate both lipid and protein substrates.^2^^,^^23^ However, the naturally occurring PTEN missense mutant G129E, which has lost its lipid phosphatase activity but retains its protein phosphatase activity, inhibits the migration of tumor cells.^24^^,^^26^ To investigate whether lipid phosphatase activity of PTEN is required for inhibition of metastasis *in vivo*, we introduced the PTEN G129E (PTEN ΔL), wildtype PTEN (PTEN WT), or the PTEN phosphatase dead mutant C124S (PTEN ΔLP) into B16F1 melanoma cells. The stable cell lines expressing the various PTEN transgenes were then tested for their metastatic potential (Figure 2C). As expected, PTEN WT significantly inhibited pulmonary metastasis *in vivo* (Figures 1F and 1G; 2A, 2B and 2C), whereas PTEN ΔLP enhanced metastasis *in vivo* by a significant degree (Figures 2A, 2B, and 2C). Surprisingly, PTEN ΔL still inhibited pulmonary metastasis to a borderline significant degree (Figure 2C). Moreover, the size of the metastases within expressing the PTEN ΔL mutant was >2 mm^3^, and bigger than those with PTEN WT, PTEN ΔLP or control (Figures 2D and S5E). These data indicate that the PTEN lipid phosphatase affects metastatic tumor growth, but is not required for suppressing metastasis *in vivo*, implying that the PTEN protein phosphatase and its lipid phosphatase activities have different roles and that the former may be more important for PTEN-mediated metastasis suppression in melanoma. Of interest, the loss of PTEN phosphatase mutation in all TCGA cancer data is a dominant mutation (Figure S6), implying that protein phosphatase is important in tumorigenesis. To verify our finding, we introduced the PTEN wildtype (WT) and various mutant form (ΔL, lipid phosphatase deficient mutant; ΔP, protein phosphatase deficient mutant; ΔLP, phosphatase dead mutant) into mouse melanoma B16F1 and rhabdomyosarcoma RMS772,^39^ did experimental metastasis studies *in vivo*. Similar to previous results, PTEN wildtype and ΔL mutant inhibited the metastasis whereas ΔLP mutant enhanced the metastasis, a protein phosphatase deficient mutant (ΔP) also promoted the metastasis (Figure S7), suggesting the protein phosphatase plays an important role in metastasis. The inactivation of PTEN protein phosphatase activity is required for promoting metastasis.

### PTEN protein phosphatase activity regulates the early survival of metastatic tumor cells

To understand how and why PTEN protein phosphatase activity regulates metastasis, we tested the biological features of stable B16F1 cells transfected with PTEN WT, ΔL and ΔLP, as well as empty vector. Overexpression of PTEN WT was found to significantly inhibit cell proliferation, motility and invasion *in vitro* in comparison to empty vector control; in contrast, clones containing the mutant forms exhibited an increase in cell proliferation and failed to inhibit cell motility (Figures 2E, 2F, and 2G). Of interest, PTEN ΔLP increased cell invasiveness, whereas PTEN ΔL had no effect (Figure 2G), supporting the notion that the lipid phosphatase activity of PTEN does not influence cell invasion *in vitro*. We also found that PTEN WT could significantly inhibit tumor growth whereas PTEN ΔL and PTEN ΔLP slightly increased tumor growth in the xenograft mouse model (Figures S5F, S5G, S5H, S5I and S5J).

**Figure 3:**
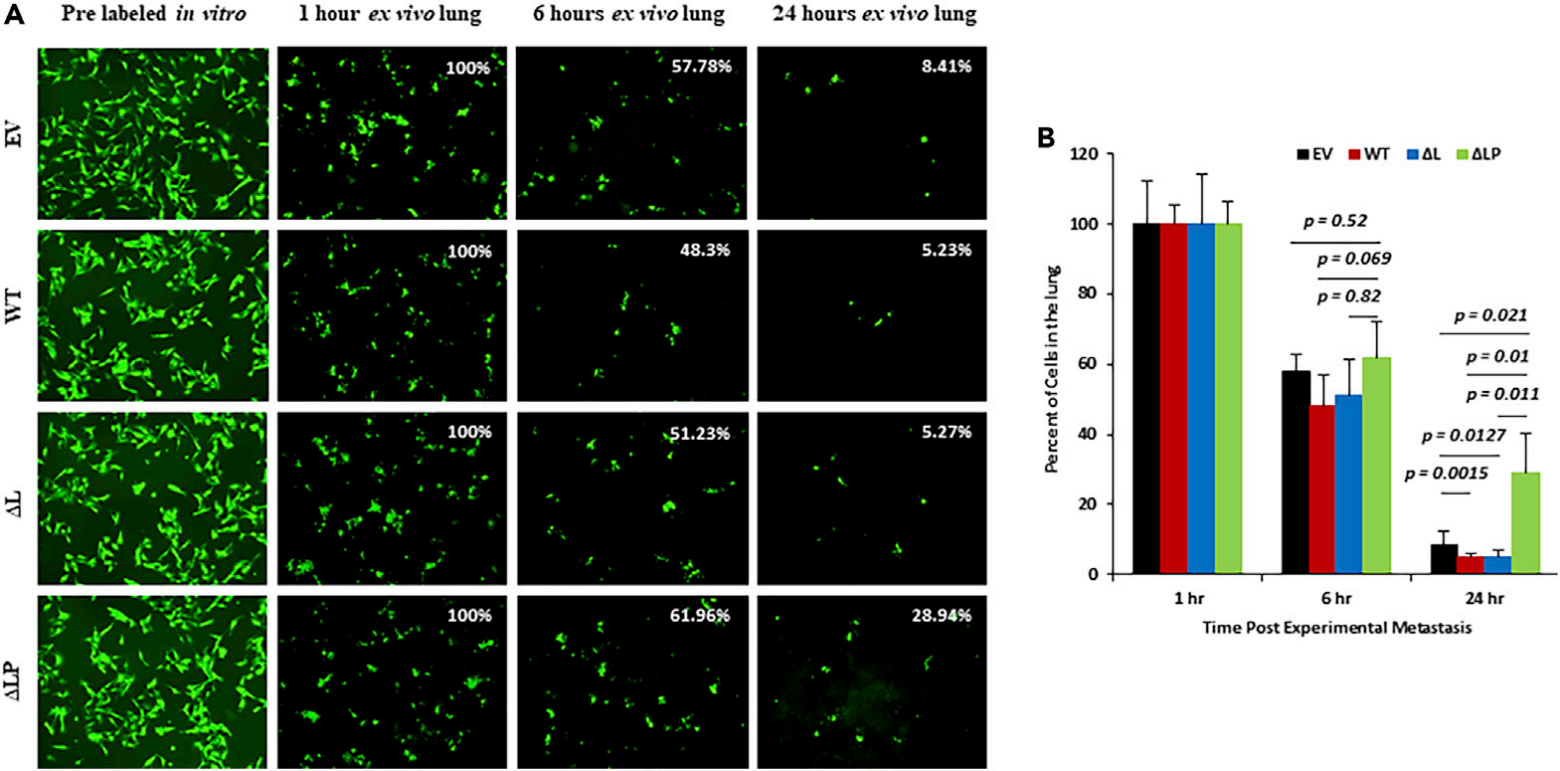
PTEN protein phosphatase inhibits early survival of tumor cells that reached the lung and mediates early metastatic survival *in vivo* (A) Images of fluorescence labeled single tumor cells arriving in the lung after experimental metastasis by the single cell-metastasis imaging system. EV, empty vector; WT, wildtype PTEN; ΔL, lipid phosphatase mutant; ΔLP, PTEN phosphatase dead mutant. (B) Quantitative data of fluorescence labeled single tumor cell arriving in lung after experimental metastasis (each group counted 5 samples). PTEN phosphatase dead mutant (ΔLP) significantly enhanced early survival of tumor cells that reached the lung after injection 24 h (ΔLP vs WT, p = 0.01; ΔLP vs ΔL, p = 0.011, no significant difference between ΔL vs WT). Data represented as mean ± SEM for all columns. The p value is shown by an unpaired t-test (two-tailed).

To further investigate the role of PTEN phosphatase activity on *in vivo* behavior, we used a single cell metastasis imaging system to track the fate of fluorescently labeled single tumor cells arriving in the lung using an experimental metastasis model. After injecting cancer cells transfected with empty vector (EV), PTEN WT, ΔL or ΔLP, the number and localization of cells arresting within the lung were defined by simultaneously imaging the fluorescent green label. One hour after tumor cells injection, the numbers of cells arrested in the lung were similar in all groups, defined as 100%. As expected, the number of cells arresting within the lung was reduced in all groups by 6 h after injection. However, although a similar fraction of remaining cells were found in the empty vector (mean 57.78%), PTEN WT (mean 48.3%) and PTEN ΔL (mean 51.23%), both were less than the rate of PTEN ΔLP (mean 61.96%). Notably, at 24 h after injection, the number of surviving cells expressing PTEN ΔLP (mean 28.94%) became 5-fold higher than cells with either PTEN WT (mean 5.23%, p = 0.01) or PTEN ΔL (mean 5.27%, p = 0.011) (Figures 3A, 3B and S5J). Our results demonstrate that PTEN protein phosphatase can hinder the survival of tumor cells reaching the lung and mediate early metastatic success. Moreover, the inactivation of its protein activity promotes the survival of tumor cells *in vivo*.

### PTEN protein phosphatase inhibits metastasis by regulating Entpd5 and IGF1R

**Figure 4:**
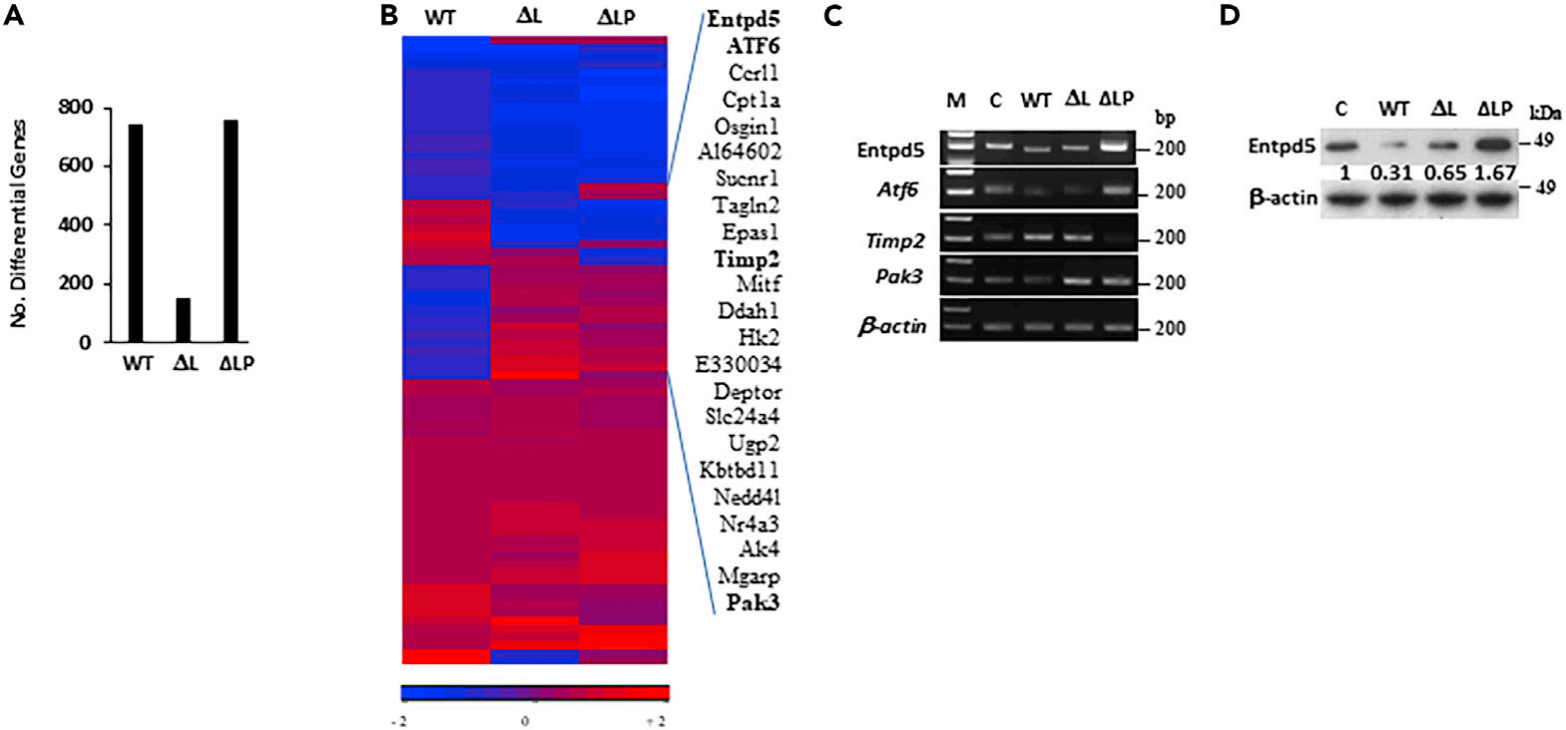
The gene expression patterns associated with melanoma progression and identification of Entpd5 as a downstream target of PTEN phosphatase (A) The significantly expressed genes in B16F1 cells with PTEN wildtype (WT), lipid phosphatase mutant (ΔL) or phosphatase dead mutant (ΔLP) forms compared with empty vector control (c) were filtered by a p value of 0.05 and absolute value of fold change of 1.5 in ANOVA analysis. (B) The common significantly altered gene list between PTEN wildtype (WT), lipid phosphatase mutant (ΔL) and phosphatase dead mutant (ΔLP) in B16F1 cells based on hierarchical clustering. (C and D) Validation of genes identified in cDNA microarray analysis by Quantitative RT-PCR (qRTPCR) (C) and Western blot analysis (D). β-actin was used as a control.

To gain more insight into the mechanism by which PTEN phosphatase regulates the metastatic process, we performed a microarray analysis of B16F1 cells expressing the various forms of PTEN. Compared to empty vector control, cells transduced with either PTEN WT (737 genes) or the PTEN ΔLP (757 genes) demonstrated alterations in the expression of a large number of genes; of interest, a much smaller number of genes (152) were altered in cells expressing the PTEN ΔL (Figures 4A and S8). Combining all groups together, we found that 76 genes were differentially altered to a significant degree (p< 0.05, Figure 4B), the majority of which were involved in the regulation of stress response, apoptosis and/or migration, including Entpd5/PCPH, Atf6, Ak4, Ugp2, Osgin1, Timp2 and Pak3. qRT-PCR and western blot were used to verify that the microarray data was an accurate representation of the gene expression patterns (Figures 4C and 4D). Entpd5 was one of the genes whose upregulation was associated exclusively with cells carrying the phosphatase-dead mutation (>2-fold in PTEN ΔLP vs.−1.6-fold in PTEN WT and −1.3 in PTEN ΔL, p < 0.05) (Figures 4B, 4C and 4D).

**Figure 5:**
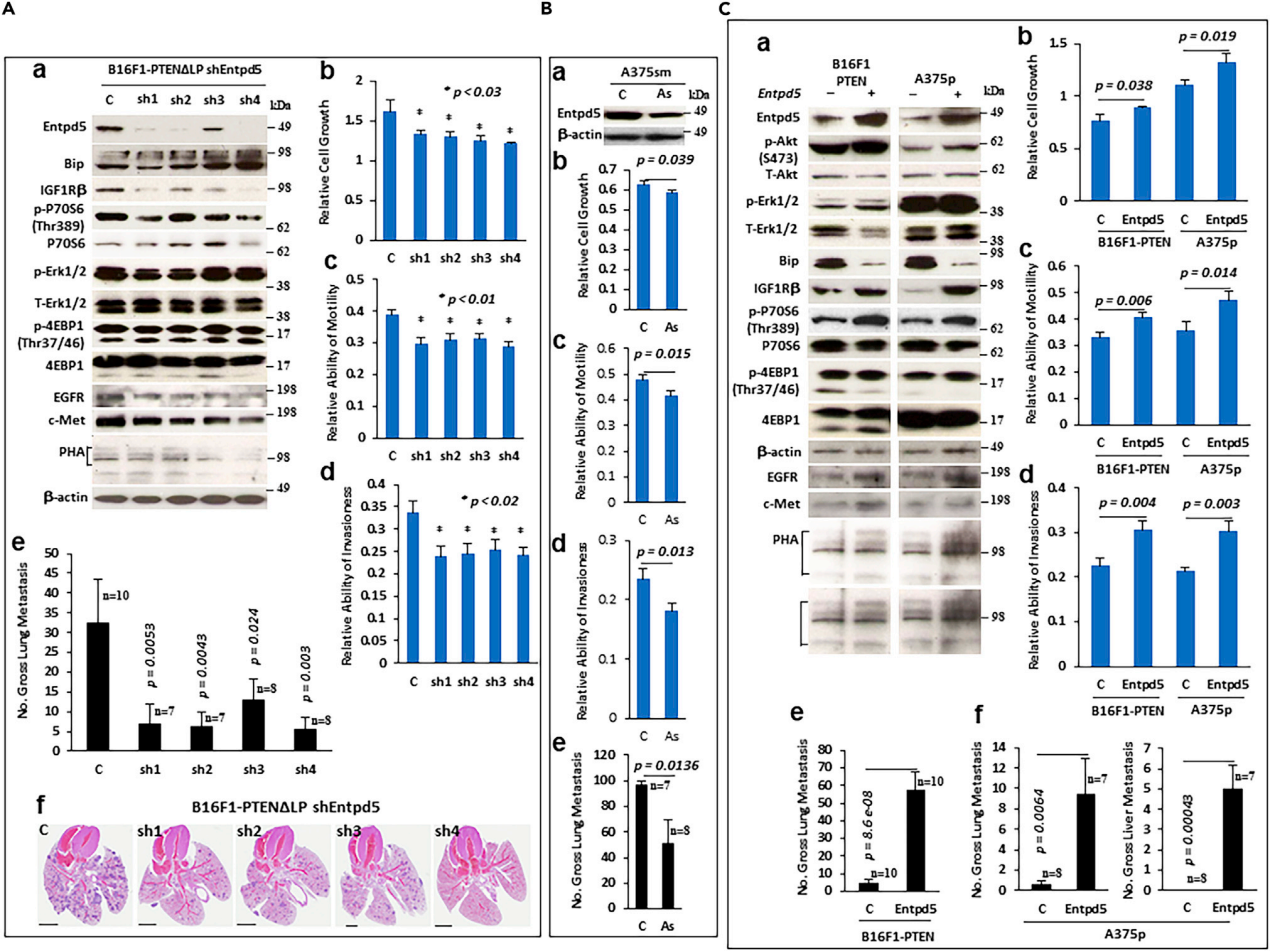
Entpd5 regulates tumor metastasis in melanoma (A) Knockdown of Entpd5 blocks PTEN phosphatase dead mutant-stimulated metastasis in melanomas. (A-a) Western blot analysis of stable B16F1-PTEN ΔLP cells transfected with shRNA plasmids for Entpd5. (A-b,c,d) Knockdown of Entpd5 affects cell growth (A-b), cell motility (A-c) and cell invasiveness (A-d) *in vitro.* Data is representative of three independent experiments. Graphs show the mean ± SEM. The p value is shown by an unpaired t-test (two-tailed). (A-e) Gross pulmonary metastases from stable B16F1-PTEN ΔLP cells transfected with shRNA plasmids for Entpd5. c, empty vector; sh1,sh2, sh3 and sh4 represent four different shRNA forms of mouse Entpd5. Data represented as mean ± SEM for all columns. The p value is shown by an unpaired t-test (two-tailed). (A-f) Representative histopathology (H&E staining) of lung sections with metastases from mice bearing B16F1-PTEN ΔLP cells transfected with shRNA plasmids for Entpd5 (scale bar = 5 mM). c, empty vector; sh1,sh2, sh3 and sh4, four different shRNA forms of Entpd5. (B) Blocking endogenous Entpd5 by antisense results in significant inhibition of metastasis. (B-a) Western blot analysis of stable A375sm cells transfected with antisense of Entpd5-expressing plasmid. (B-b, c, d) Alteration of cell growth (B-b), motility (B-c) as well as invasiveness (B-d) *in vitro* in human A375sm cells transfected with antisense of Entpd5 expressing plasmid. Data is representative of three independent experiments. Graphs show the mean ± SEM. The p value is shown by an unpaired t-test (two-tailed). (B-e) Gross pulmonary metastases from A375sm cell transfected with Entpd5 antisense. c, empty vector; As, antisense for Entpd5. Data represented as mean ± SEM for all columns. The p value is shown by an unpaired t-test (two-tailed). (C) Overexpression of Entpd5 abrogates PTEN wildtype-mediated metastatic suppression. (C-a) Western blot analysis of stable B16F1-PTEN WT and A375p cell lines transfected with an Entpd5-expressing plasmid. (C-b, c, d) Forced expression of Entpd5 stimulates cellular proliferation (C-b), motility (C-c) and invasiveness (C-d) *in vitro*. Data is representative of three independent experiments. Graphs show the mean ± SEM. The p value is shown by an unpaired t-test (two-tailed). (C-e, f) Gross pulmonary or liver metastases from B16F1-PTEN and A375p cells transfected with an Entpd5 expressing vector. – or c, empty vector; + or Entpd5; Entpd5 expressing vector. Data represented as mean ± SEM for all columns. The p value is shown by an unpaired t-test (two-tailed).

Entpd5 was previously found to be upregulated in PTEN deficient MEF cells.^40^ We hypothesized that Entpd5 plays an important role in PTEN-mediated regulation of metastasis, specifically through PTEN protein phosphatase activity. To study this, we first used shRNAs to knockdown Entpd5 in PTEN ΔLP cells (Figure 5A-a) to determine the effects on *in vitro* proliferation, motility and invasiveness, as well as *in vivo* metastasis. Consistent with previous studies,^40^ downregulation of Entpd5 could affect the glycolysis of receptor tyrosine kinase and reduce the expression of IGF1R and EGFR as well as c-Met (Figure 5A-a). Also, knockdown of Entpd5 in B16F1 cells expressing PTEN ΔLP significantly blocked pulmonary metastasis by a factor of 3-fold–5-fold following tail vein injection into syngeneic mice (*p* = from 0.024 to 0.003, Figure 5A-e, f). Moreover, downregulation of endogenous Entpd5 by transfection of human Entpd5 antisense in highly metastatic human A375sm melanoma cells, characterized by a lower level of PTEN and higher Entpd5, also reduced pulmonary metastasis significantly when cells were introduced into NSG mice by orthotopic footpad injection (p = 0.0136, Figures 5B-e, S9A). Downregulation of Entpd5 also significantly inhibited cell proliferation and invasion in both cell lines and reduced their motility (Figure 5A-b, c, d; B-b, c, d).

To further elucidate the role of Entpd5 in PTEN-suppressed metastasis, Entpd5 was overexpressed in poorly metastatic mouse B16F1-PTEN WT and human melanoma A375p cells, where expression levels of Entpd5 are relatively low and PTEN relatively high (Figure 5C-a). Both cell lines were tested for their ability to metastasize when introduced into C57BL/6-cBrd mice by tail vein injection (B16F1-PTEN WT) and into NSG mice by orthotropic footpad injection (A375p). Forced expression of Entpd5 significantly stimulated pulmonary metastasis in both cell lines (p = 8.6e-0.8 and p = 0.0064, respectively) and liver metastasis in A375p (p = 0.00043) (Figures 5C-e, f; S9B, S9C and S9D). Moreover, overexpression of Entpd5 significantly increased cell proliferation, motility and invasiveness in both cell lines (in B16F1, p = 0.038, p = 0.006 and p = 0.004 respectively; in A375p, p = 0.019, p = 0.014 and p = 0.003 respectively; Figure 5C-b,c,d).

**Figure 6:**
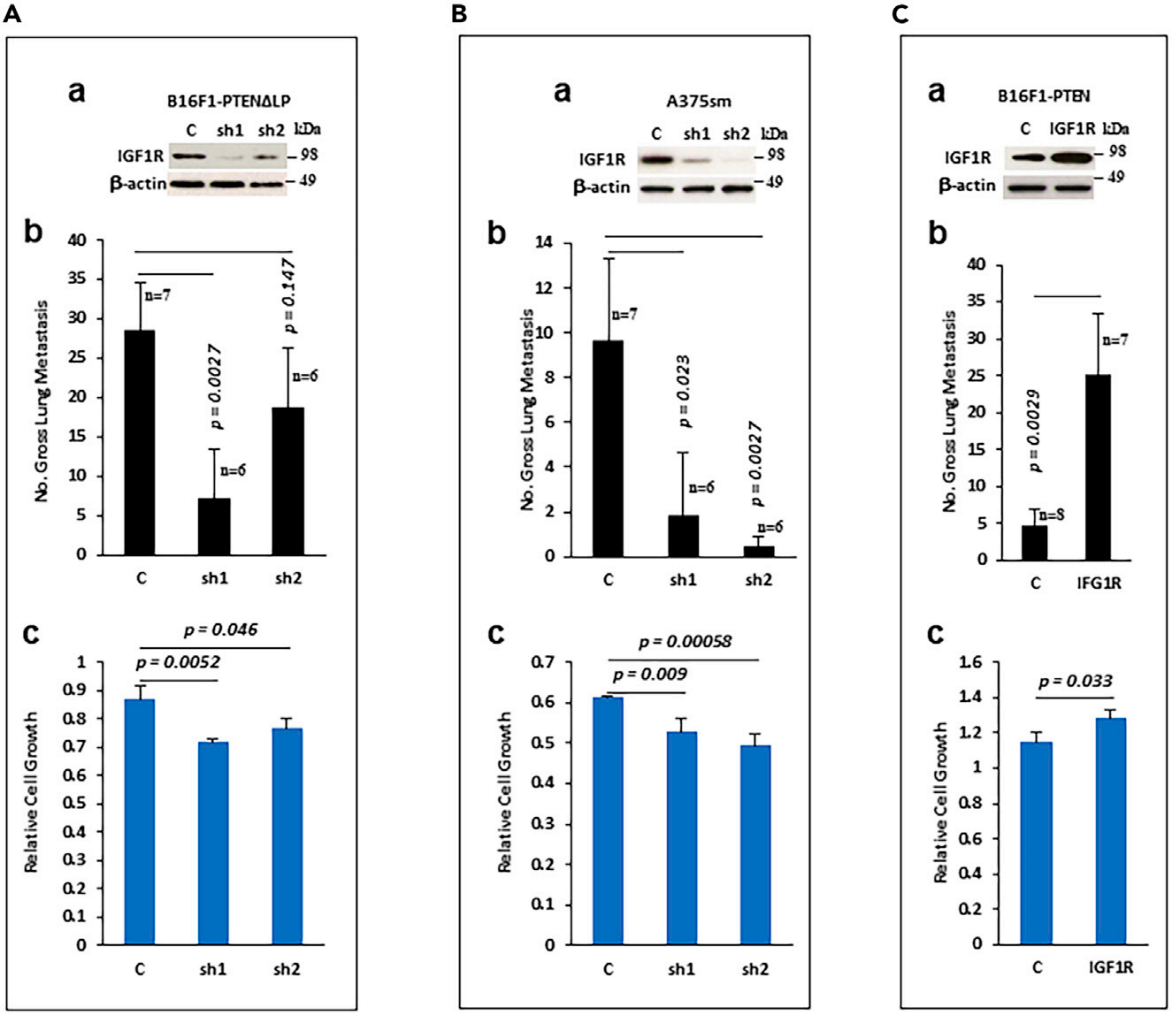
IGF1R regulates tumor metastasis in melanoma cells (A) Knockdown of IGF1R expression also blocks PTEN phosphatase dead mutant-stimulated metastasis. (A-a) Western blot analysis of stable B16F1-PTEN ΔLP cells transfected with shRNA plasmids for mouse IGF1R. (A-b) Gross pulmonary metastases from B16F1-PTEN ΔLP cells transfected with shRNA for mouse IGF1R. Data represented as mean ± SEM for all columns. The p value is shown by an unpaired t-test (two-tailed). (A-c) Effects on cell growth by shRNA of IGF1R. Data is representative of three independent experiments. Graphs show the mean ± SEM. The p value is shown by an unpaired t-test (two-tailed). c, empty vector; sh1 and sh2, shRNA expression plasmid for mouse IGF1R. (B) Knockdown of IGF1R expression inhibits metastasis in human melanoma A375sm cells. (B-a) Western blot analysis of stable A375sm cells transfected with shRNA plasmids for human IGF1R. (B-b) Gross pulmonary metastases from transfected cells. Data represented as mean ± SEM for all columns. The p value is shown by an unpaired t-test (two-tailed). (B-c) Effects on cell growth by shRNAs of IGF1R. Data is representative of three independent experiments. Graphs show the mean ± SEM. The p value is shown by an unpaired t-test (two-tailed). c, empty vector; sh1 and sh2, shRNA expressing plasmid for human IGF1R. (C) Forced expression of IGF1R results in the abrogation of PTEN-mediated inhibition of metastasis. (C-a) Western blot analysis of stable B16F1-PTEN cells transfected with IGF1R expressing plasmid. (C-b) Gross pulmonary metastases from transfected cells. Data represented as mean ± SEM for all columns. The p value is shown by an unpaired t-test (two-tailed). (C-c) Effects on cell growth by IGF1R. Data is representative of three independent experiments. Graphs show the mean ± SEM. The p value is shown by an unpaired t-test (two-tailed). c, empty vector; IGF1R, IGF1R expressing plasmid.

Entpd5 encodes an ER UDPase whose activity promotes the N-glycosylation and folding of proteins, including receptor tyrosine kinases such as IGF1R. Entpd5 can promote IGF1R trafficking to the cell membrane, thereby regulating its abundance and signaling activity.^40^^,^^41^ We found that knockdown of Entpd5 in B16F1 PTEN ΔLP cells significantly reduced IGF1R levels (Figure 5A-a). Overexpression of Entpd5 in both B16F1-PTEN WT and A375p cells increased levels of IGF1R (Figure 5C-a). To further explore the involvement of IGF1R in PTEN-mediated metastasis suppression, we knocked down IGF1R in mouse B16F1-PTEN ΔLP cells (Figure 6A-a) and human A375sm cells (Figures 6B-a), where expression levels of IGF1R and Entpd5 are relatively high, and then tested their ability to metastasize by tail vein injection (B16F1-PTEN ΔLP) or orthotropic footpad injection (A375sm). Successful disruption of IGF1R expression in both cell lines significantly inhibited cell proliferation (Figures 6A-c and 6B-c) and pulmonary metastasis *in vivo* (Figures 6A-b, S9E, B-b, and S9F). Conversely, overexpression of IGF1R in B16F1-PTEN WT cells (Figure 6C-a) significantly stimulated cell proliferation (Figure 6C-c) and pulmonary metastasis (Figures 6C-b, S9G). These data indicate that IGF1R is involved in the regulation of PTEN-mediated suppression of metastasis. Taken together, our results demonstrate that PTEN protein phosphatase can inhibit metastasis by negatively regulating the Entpd5/IGF1R pathway.

### PTEN expression inversely correlates with Entpd5 and IGF1R expression in human melanoma samples

**Figure 7:**
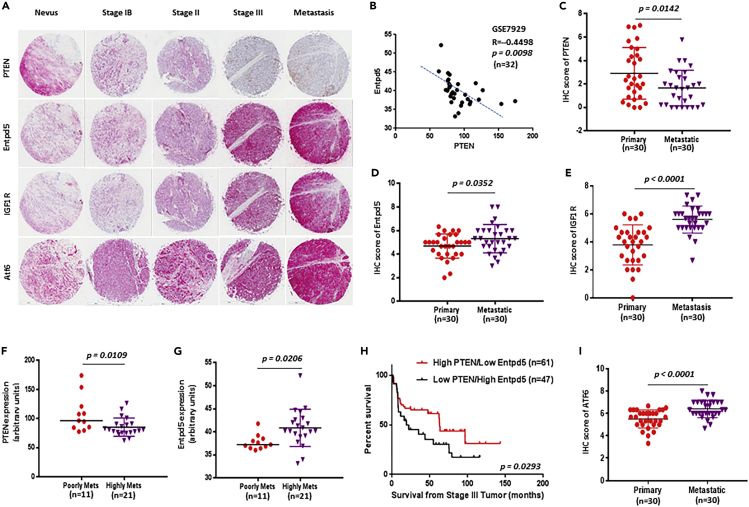
PTEN expression negatively correlates with Entpd5 and IGF1R in human melanoma samples (A) Immunohistochemistry analyses of tissue microarrays were performed using antibodies against PTEN, Entpd5 and IGF1R in consecutively cut sections of nevi (n = 18), melanomas (n = 56) (stage IB, n = 6; Stage II, n = 26; Stage III, n = 6, other, n = 18) and metastatic melanomas (n = 26). Representative examples are shown of tumor cores with high-, medium-, low- or none-intensity PTEN staining, with their corresponding intensities of Entpd5 and IGF1R (low-, medium- or high-intensity staining). Scale bar = 20 μM. (B) Representative example showing a significantly negative correlation between PTEN and Entpd5 at the transcription level in the GSE7929 dataset by Pearson r (two-tailed) analysis. (C-E and I) PTEN (C), Entpd5 (D), IGF1R (E) and ATF6 (I) expression was determined by immunohistochemistry using antibodies against PTEN, Entpd5, IGF1R or ATF6 in tissue microarray with 30 melanomas and 30 metastatic melanomas. They were scored for a combination of staining intensity on a 0–3 scale (none =0, low = 1, medium = 2 and high = 3) and a frequency of tumor labeling on a 0–5 scale (no cells = 0, <10% = 1, 10–32% = 2, 33–65%= 3, 66–99% = 4 and 100% = 5). (F, G) Expression of PTEN and Entpd5 in poorly and highly metastatic melanoma tumors in the GSE7929 dataset. Data represented as mean ± SEM. The p value is shown by an unpaired t-test (two-tailed). (H) Analyses of GSE53118 and GSE54467 late-stage melanoma datasets show that high PTEN with low Entpd5 expression is associated with a longer time of survival in late-stage malignant melanoma [p = 0.0293 by Logrank (Mantel-Cox) test].

Because we observed that Entpd5 expression was decreased in melanoma cells overexpressing WT PTEN and increased in melanoma cells expressing phosphatase-deficient PTEN, we sought to examine the relationship between PTEN, Entpd5 and IGF1R expression in human melanomas by immunohistochemistry using serial tissue microarray sections. Figure 7A shows that the samples with higher intensity PTEN staining had lower intensities of Entpd5 and IGF1R; conversely, the samples with lower or loss of intensity of PTEN staining exhibited higher intensities of Entpd5 or IGF1R. Pearson R analysis of these data showed that the negative correlation of PTEN staining with both Entpd5 and IGF1R was statistically significant (Figures S10A and S10B). However, there was a positive correlation between Entpd5 and IGF1R expression (Figure S10C). Moreover, we also found an inverse correlation between PTEN and both Entpd5 and IGF1R expression and a positive correlation between Entpd5 and IGF1R in the GSE7929 (Figure 7B), GSE53118 (Figures S11A–S11C), GSE54467 (Figures S12A–S12C), and GSE22138 (Figure S13) datasets. To determine if the observed relationship between PTEN/Entpd5 and metastasis holds up in clinical samples, we screened a panel of primary melanoma and metastatic melanoma tissues for the expression of PTEN and Entpd5 protein by immunohistochemical staining. Compared with primary melanomas, PTEN expression was significantly decreased or lost in metastatic melanomas (p = 0.0142, Figure 7C); in contrast, Entpd5 and IGF1R expression was increased significantly in metastatic melanomas (p = 0.0352 and p < 0.0001 respectively Figures 7D and 7E). We also observed that levels of PTEN transcripts were significantly reduced in highly metastatic samples compared to poorly metastatic melanomas, whereas Entpd5 transcripts were elevated in highly metastatic melanomas (GSE7929 dataset; Figures 7F and 7G). Notably, an analysis of datasets with available information on both metastasis and patient survival (GSE53118 and GSE54467; Stage 3 melanomas) demonstrated that patients with high PTEN/low Entpd5 expression had a significantly better outcome relative to patients with low PTEN/high Entpd5 (Figures 7H, S11D, S11E, S12D, and S12E). Our results demonstrate that PTEN expression negatively correlates with Entpd5 and IGF1R, supporting the notion that the PTEN/Entpd5/IGF1R pathway plays an important role in regulating melanoma metastasis.

### Inactivated PTEN associates with Entpd5 expression through activating ATF6

**Figure 8:**
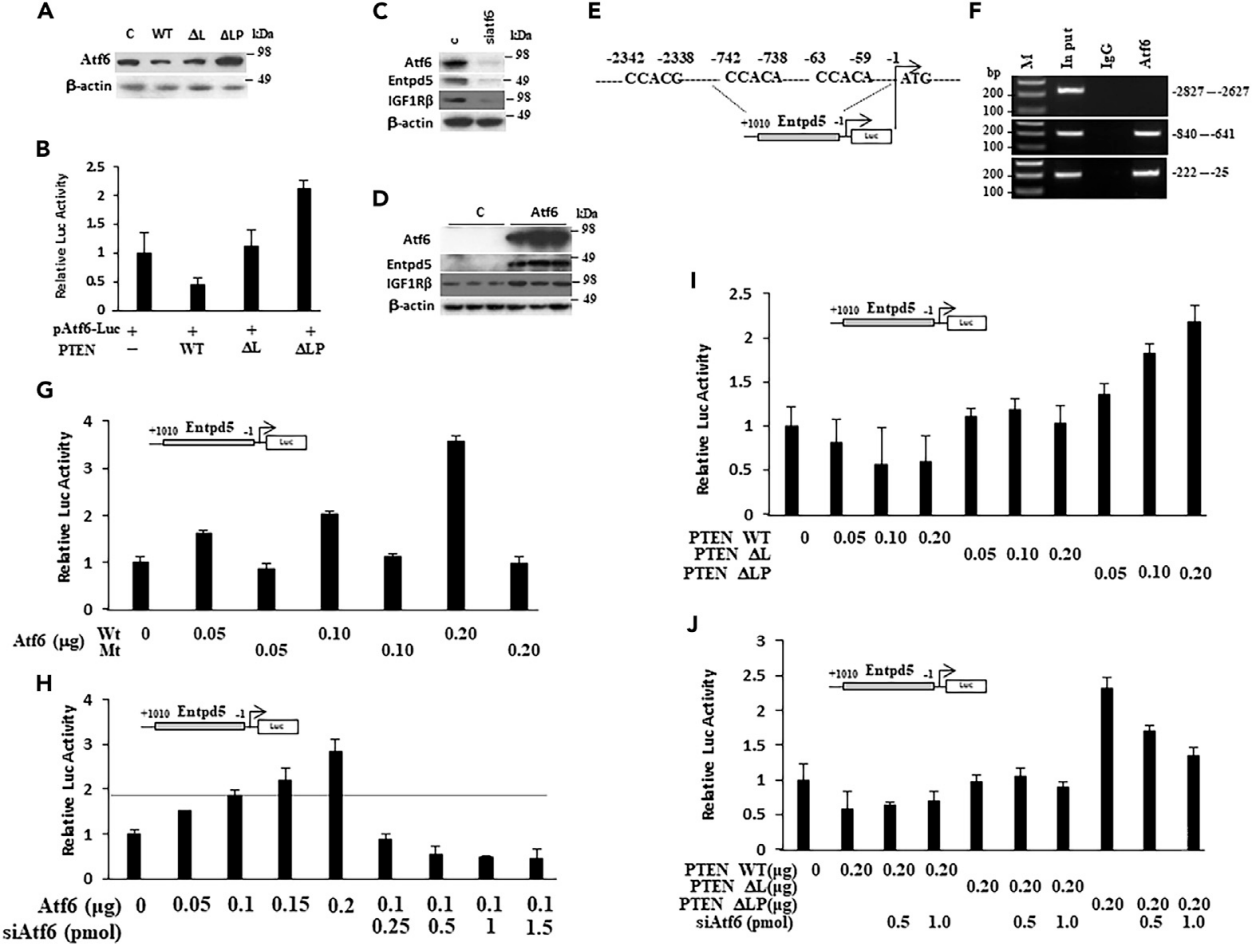
ATF6 regulated by PTEN can directly bind to the Entpd5 promoter and regulate the expression of Entpd5 (A) ATF6 protein expression was analyzed by western blot in cells bearing various PTEN mutants. PTEN WT expression decreased ATF6 expression, whereas PTEN ΔLP increased ATF6 expression. (B) Effects of PTEN mutants on 5xATF6 binding site-driven luciferase activity. Data is representative of three independent experiments. Graphs show the mean ± SEM. (C and D) Entpd5 and IGF1R protein expression was analyzed by western blot in B16F1 cell lines transfected with either an ATF6 expression vector or treated with siRNA for ATF6. Knockdown of ATF6 through an RNAi mechanism inhibited Entpd5 expression in B16F1-PTEN ΔLP (C), whereas ectopic ATF6 expression stimulated Entpd5 expression in B16F1-PTEN (D). c, empty vector control. (E) Three candidate ATF6 binding sites consisting of the 5′-CCAC[GA]-3′ half of the ER stress response element (ERSE) were found within the 5′-flanking region (between −2342 and -1bp) of the Entpd5 promoter. (F) The physical interaction of ATF6 to Entpd5 gene promoter was assessed by ChIP assay. Native ATF6 in B16F1-PTEN ΔLP cells was analyzed using an anti-ATF6 antibody. ATF6 bound to the −840 to −641 and −222 to −25 regions of the Entpd5 promoter, but not the −2827 to −2627 regions. Inp, input; M, markers. (G), Entpd5 gene promoter (−1010 to −1) activity was shown to be responsive to increasing amounts of an ATF6 expression vector using a firefly luciferase (Luc) reporter, while not to be responsive to an ATF6 mutant form that lost its binding activity. (H) Using the same luciferase assay, the addition of siRNA for ATF6 with an ATF6-expression vector inhibited luciferase activity driven by the Entpd5 promoter. (I) Entpd5 gene promoter (−1010 to −1) activity was shown to be stimulated by increasing amounts of a PTEN ΔLP expression vector using a firefly luciferase (Luc) reporter, while not to be responsive to PTEN ΔL. (J) Using the same luciferase assay, addition of siRNA for ATF6 inhibited PTEN ΔLP-stimulated luciferase activity driven by the Entpd5 promoter in a co-transfection assay. Data is representative of three independent experiments. Graphs show the mean ± SEM.

Our gene expressing profile showed that the transcription factor ATF6, which is a critical ER stress sensor and initiator of the ‘unfolded protein response’ (UPR),^42^ was also elevated in PTEN phosphatase-dead mutant cells (Figure 4B). To further understand the biological connection between PTEN and Entpd5/IGF1R pathway, we first assessed the expression levels of ATF6 in melanoma cells carrying the various PTEN mutants by western blot (Figure 8A); ATF6 expression was reduced in PTEN WT cells, but enhanced in PTEN ΔLP cells. When the various PTEN mutants were co-transfected into B16F1 cells with a luciferase reporter driven by 5x ATF6 binding sites, the PTEN phosphatase dead mutant increased luciferase activity whereas PTEN WT decreased luciferase activity, and there was no change in PTEN ΔL cells compared with empty vector control (Figure 8B). These results suggest that ATF6 is regulated by PTEN and upregulates Entpd5 expression. To test this hypothesis, the introduction of siRNA for ATF6 in B16F1 PTEN ΔLP cells resulted in downregulation of Entpd5 as well as IGF1R (Figure 8C); conversely, overexpression of ATF6 in B16F1 PTEN WT cells upregulated Entpd5 and IGF1R (Figure 8D). These data indicate that ATF6 can regulate Entpd5 expression, and raise the possibility that this ER stress response factor transcriptionally activates the gene encoding Entpd5. Really, the expression of ATF6 is correlated with Entpd5 expression in SKCM-TCGA data (Figure S14). We also examined the ATF6 expression in human melanoma by immunohistochemistry using serial tissue microarray sections compared with PTEN, Entpd5 and IGF1R (Figure 7A), and found that the ATF6 expression negatively correlated with PTEN expression (Figure S10D), while positively with Entpd5 (Figure S10E) and IGF1R (Figure S10F). Moreover, we found that ATF6 expression was increased significantly in metastatic melanomas after screening a panel of primary melanoma and metastatic melanoma tissues for the expression of ATF6 in a serial tissue microarray (same as PTEN and Entpd5 and IGF1R) by immunohistochemical staining (p< 0.001, Figure 7I).

To determine if ATF6 directly binds to the Entpd5 gene promoter in chromatin, a ChIP assay was performed. Three candidate ATF6 binding sites consisting of the 5′-CCAC[GA]-3′ half of the ER stress response element (ERSE) were found within the 5′-flanking region (between −2342 and -1bp) of the Entpd5 promoter (Figures 8E). Figure 8F shows that ATF6 can bind to the Entpd5 promoter between −840 and−641 and −222 to −25 regions containing CCACA sequence, but not to an irrelevant site between −2827 and −2627. To functionally test the activity of ATF6 on the Entpd5 promoter, we cloned the Entpd5 promoter region between −1010 and −1 to establish an Entpd5 promoter-luciferase reporter construct and determined the effect of ATF6 on the luciferase activity. Figure 8G shows that ATF6 can stimulate luciferase activity in B16F1 cells harboring an Entpd5 promoter-luciferase expression vector in a dose-dependent fashion, an effect that can be reversed with an ATF6 siRNA (Figure 8H). Moreover, an inactive non-binding ATF6 mutant failed to stimulate luciferase activity in the same cells (Figure 8G). To further confirm the role of ATF6 as a link between PTEN and Entpd5, we co-transfected various PTEN mutants with the Entpd5 promoter-luciferase reporter vector into B16F1 cells. As shown in Figure 8I, PTEN ΔLP could stimulate the Entpd5 promoter-driven luciferase activity in a dose-dependent fashion, but not PTEN WT or PTEN ΔL. Notably, this PTEN ΔLP-stimulated luciferase activity was blocked by siRNA-ATF6 (Figure 8J). Taken together, our data show that ATF6, which is overexpressed in the absence of PTEN protein phosphatase activity, directly binds to the Entpd5 promoter and transcriptionally regulates Entpd5 expression.

## Discussion

PTEN is frequently mutated in advanced malignant diseases, and the reduction of PTEN protein levels, either through genetic or epigenetic mechanisms, has been correlated with susceptibility in various human cancers.^3^^,^^4^^,^^43^^,^^44^ GEM models have generally supported this clinical relationship: Engineered reduction of PTEN causes enhanced tumor susceptibility and incidence,^10^^,^^11^ whereas PTEN overexpression augments its tumor-suppressive function and protects from tumorigenesis,^12^ strongly suggesting that PTEN levels are associated with tumor initiation and progression.^8^^,^^9^^,^^10^^,^^11^^,^^43^^,^^44^^,^^45^ However, much less is known about the role of PTEN protein levels in metastasis or the mechanisms and pathways that might link them. In melanoma, a few studies have provided data suggesting that metastasis is associated with the inactivation of PTEN.^16^^,^^35^^,^^46^^,^^47^

In this study, we show that PTEN expression correlates with progression to the metastatic state in human melanomas, and provide an experimental link using an HGF-transgenic, PTEN-deficient mouse melanoma model. We also assess the effects of expressing PTEN and its various phosphatase-crippled mutants on melanoma metastasis and show that PTEN can regulate metastasis in a dose-dependent manner and function as a haploinsufficiency tumor suppressor. We demonstrate for the first time that metastasis is controlled by PTEN through inhibition of the protein N-glycosylation and folding promoter Entpd5, reducing IGF1R levels through the regulation of ATF6. Notably, this inhibition requires PTEN’s protein phosphatase activity, but not its lipid phosphatase activity.

In accord with our findings, previous studies have provided strong evidence that the anti-migration activities of PTEN are not dependent on its lipid phosphatase activity.^24^^,^^26^ The PTEN lipid phosphatase mutant was found to inhibit the invasion of human bladder cancer cells to a similar degree as WT PTEN.^48^ PTEN does not require its lipid phosphatase activity to control DNA repair and sensitivity to genotoxic stress.^27^ PTEN was recently reported to serve as a protein tyrosine phosphatase for IRS1 through its protein phosphatase activity,^49^ implicating this activity in cell migration. And although re-expression of a specific protein phosphatase-deficient mutant also could inhibit cell migration of PTEN-deficient U87MG glioblastoma cells in a wound-healing assay, PTEN WT and lipid phosphatase deficient could inhibit migration.^50^^,^^51^ That the PTEN lipid phosphatase is not required for inhibiting metastasis is consistent with all these observations, whereas the PTEN phosphatase dead mutant could enhance metastatic potential, suggests that the PTEN protein phosphatase activity may play an important role in inhibiting metastasis. The two phosphatases affect different tumor phenotypes. Our data indicate that the lipid phosphatase could inhibit melanoma growth, whereas the protein phosphatase regulated motility and invasiveness. It is worth noting that the melanoma cell lines we used as model systems express endogenous WT PTEN, consistent with the notion that PTEN mutants may function in a dominant-negative fashion.^38^

The mechanism by which PTEN regulates metastasis is not clear. Reported studies have proposed that the PTEN protein phosphatase activity might dephosphorylate FAK, thereby reducing cell migration.^24^^,^^25^^,^^52^ However, we could not find any change in FAK activity in our melanoma cells associated with PTEN phosphatase activity (data not shown); suggesting that other molecules or pathways may be involved. Through microarray analysis of cells carrying various PTEN mutants, we have found that two ER proteins, Entpd5 and ATF6, were expressed at significantly higher levels in PTEN phosphatase dead mutant cells and lower levels in cells with PTEN WT compared with empty vector control cells. Entpd5 is an ER UDPase whose activity hydrolyzes UDP to UMP to promote glycosylation and folding of glycoproteins in ER.^41^ It has been reported that the expression of Entpd5 in several types of cancers resulted in altered cellular metabolism, increased survival and invasiveness as well as metastasis, and enhanced resistance to stress stimulators.^53^^,^^54^ Entpd5 expression was also highly increased in PTEN-null cells, resulting in ER stress relief, increased IGF1R and enhanced survival.^40^ Our data showed that knockdown of Entpd5 in cells expressing the PTEN phosphatase dead-mutant blocked its ability to promote metastasis, reduced IGF1R and promoted cellular ER stress. In contrast, overexpression of Entpd5 in cells expressing PTEN WT abrogated PTEN’s ability to inhibit metastasis, enhance IGF1R and inhibit cell ER stress.

To be metastatically successful tumor cells must overcome a state of chronic ER stress caused by nutrition restriction, hypoxia, and/or altered metabolism. ER stress is triggered by impaired protein synthesis, increased levels of unfolded protein, and the resulting enhanced homeostatic unfolded protein response (UPR).^55^ Activated UPR signaling induces apoptotic pathways by the ER stress master regulator BiP/Grp78 and three sensors: ER-to-nucleus signaling 1 (IRE1a), protein kinase RNA-like ER kinase (PERK), and ATF6.^56^ ER stress-activated ATF6 is transferred into the nucleus where it modulates the expression of select genes. ATF6 has been identified as a survival factor in cancer cells and is associated with liver carcinogenesis^57^ and tumor dormancy.^58^ We here show that ATF6 could directly bind to the Entpd5 gene promoter and regulate its expression, linking the UPR signaling-folding protein promoter Entpd5 to cell survival and metastasis of melanoma cells.

In fact, melanoma and other cancer cells are largely resistant to ER stress-induced apoptosis, suggesting that the kinetics and duration of activation of UPR pathways are deregulated in melanoma cells undergoing ER stress.^59^ We propose that PTEN mutation or inactivation is one means by which tumor cells become resistant to ER stress-induced apoptosis, resulting in increased ATF6 and enhanced Entpd5/IGF1R expression, promoting protein glycosylation and folding. Indeed, the expression of a PTEN phosphatase mutant promoted the early survival of metastatic cells *in vivo*.

In summary, our studies provide evidence that loss of PTEN phosphatase activity is a critical step in the progression of metastatic disease. Expression of PTEN helps determine metastatic behavior in a dose-dependent manner, and patients with lower PTEN and higher Entpd5 levels might represent a population at high risk (susceptibility) to developing the metastatic disease; these proteins may prove to be valuable biomarkers for predicting metastatic potential. Moreover, our data indicate that the PTEN protein phosphatase is important for PTEN-mediated suppression of metastasis, whereas its lipid phosphatase is primarily responsible for the growth of melanoma. Our findings provide mechanistic insight into the regulation of metastatic potential by PTEN phosphatase and demonstrate a link between PTEN and ER stress activation in metastasis. Knowledge of the status of the PTEN/ATF6/Entpd5/IGF1R axis may prove to be valuable in the design of novel preventive and interventive therapeutic strategies in melanoma.

### Limitations of the study

Although we found overexpression of wildtype PTEN downregulated the ATF6 expression whereas phosphatase-mutant PTEN upregulated the ATF6 protein level, moreover; when the various PTEN mutants were co-transfected into B16F1 cells with a luciferase reporter driven by 5x ATF6 binding sites, the PTEN phosphatase dead mutant increased luciferase activity whereas PTEN WT decreased luciferase activity, and there was no change in PTEN ΔL cells compared with empty vector control. These results suggest that PTEN phosphatase activity regulates ATF6. However, we have not addressed a direct link between PTEN and ATF6 and how PTEN regulates ATF6 expression through its protein phosphatase activity. Recent studies reported that PTEN could interact with many transcriptional factors, such as AFF4, RNAPII, CDK9, cyclin T1, XPB, and CDK7,^60^ and its protein phosphatase activity could dephosphorylate some transcriptional factors such CREB, MCM2, and NKX3.1 and RNA polymerase II,^61^^,^^62^^,^^63^ therefore regulate the gene transcription. Moreover, PTEN phosphorylation at its C-terminal (C-tail) serine/threonine cluster negatively regulates its tumor suppressor function.^26^^,^^63^ Replacement of the serine/threonine residues with alanine-generated PTEN-4A, a phosphorylation-deficient PTEN mutant, preferentially localized to the nucleus, where it suppressed E2F1-mediated transcription of cell cycle genes.^64^ The study also showed that phosphorylated PTEN at S380 under stress conditions affected PTEN nuclear localization and blocking of PTEN nuclear function.^65^ With our results, because of PTEN protein phosphatase activity could dephosphorylate PTEN itself at S380,^26^^,^^63^ we speculate that PTEN protein phosphatase activity may affect PTEN nucleolus localization and interaction with other proteins, such as RNA polymerase, to bind to gene promoters and then regulate gene transcription such as ATF6 indirectly. The expanded studies will be critical in the future to determine how PTEN could indirectly regulate the ATF6 transcription.

## STAR★Methods

### Key resources table

**Table:** 

REAGENT or RESOURCE	SOURCE	IDENTIFIER
**Antibodies**		

anti-PTEN antibody	CASCADE biosciences	Cat# ABM-2052, RRID:AB_2335636
anti-phospho-AKT (Ser473)	Cell Signaling	Cat# 4060, RRID:AB_2315049
anti-phospho-AKT (Thr308)	Cell Signaling	Cat# 13038, RRID:AB_2629447
anti-AKT	Cell Signaling	Cat# 4691, RRID:AB_915783
anti-phospho-ERK 1/2 (Thr202/Tyr204)	Cell Signaling	Cat# 4370, RRID:AB_2315112
anti-ERK1/2	Cell Signaling	Cat# 4695, RRID:AB_390779
anti-IGF1Rβ	Cell Signaling	Cat# 9750, RRID:AB_10950969
anti-GSK3α	Cell Signaling	Cat# 4337, RRID:AB_10859910
anti-phospho-GSK3α(Ser21)	Cell Signaling	Cat# 9316, RRID:AB_659836
anti-GSK3β	Cell Signaling	Cat# 12456, RRID:AB_2636978
anti-phospho-GSK3β (Ser9)	Cell Signaling	Cat# 5558, RRID:AB_10013750
anti-p70S6	Cell Signaling	Cat# 2708, RRID:AB_390722
anti-phospho-p70S6 (Thr389)	Cell Signaling	Cat# 9205, RRID:AB_330944
anti-4EBP1	Cell Signaling	Cat# 9644, RRID:AB_2097841
anti-phospho-4EBP1 (Thr37/46)	Cell Signaling	Cat# 2855, RRID:AB_560835
anti-PTEN	Cell Signaling	Cat# 9559, RRID:AB_390810
Anti-IGF1R	Sigma-Aldrich	Cat# SAB4300359, RRID:AB_10622734
anti-Atf6	Abcam	Cat# ab227830
anti-Entpd5	Abcam	Cat# ab108603, RRID:AB_10862496
anti-EGFR	Millipore	Cat# SAB4300454
anti-atf6	Thermo Scientific	Cat# MA5-16172, RRID:AB_2537691
anti-Entpd5	Thermo Scientific	Cat# PA5-29503, RRID:AB_2546979
anti-Met	Santa Cruz	Cat# sc-8057, RRID:AB_673755
anti-β-actin	Santa Cruz	Cat# sc-81178, RRID:AB_2223230
PHA-E	US Biological	Cat# P3370-27A
ENTPD5/CD39L4 Antibody (IHC)	LSBio	Cat# LS-B8926
PTEN Antibody (clone 6H2.1, IHC)	LSBio	Cat# LS-C743530
ATF6 Antibody (IHC)	LSBio	Cat# LS-B2430
IGF1R/IGF1 Receptor Antibody (clone 1H7, IHC)	LSBio	Cat# LS-B2613
ATF6 siRNA	GE Dharmacon	L-044894-00-0005
Non-targeting control siRNA	GE Dharmacon	D-001810-10-05

**Chemicals, peptides, and recombinant proteins**		

Lipofectamine 2000 reagent	Invitrogen	Cat# 11668019
Antibiotics G418	Sigma	Cat# A1720-5G
Puromycin	Sigma	Cat# P9620
Green CMFDA	Invitrogen	Cat# C7025

**Critical commercial assays**		

Tissue microassay analysis: Human ME207, ME208, ME1004b	US Biomax	

**Deposited data**		

Microarray data	This paper	GSE210359

**Experimental models: Cell lines**		

Mouse: B16F1	ATCC	Cat# CRL-6323
Mouse: 37-7	Yu Y, et al.^37^	Cancer Res 2002
Mouse: RMS772	Yu Y, et al.^39^	Nat Med 2004
Human: A375p	M.D. Anderson Medical Center	gift from Dr. Isaiah Fidler
Human: A375sm	M.D. Anderson Medical Center	gift from Dr. Isaiah Fidler
Human: A375c5	M.D. Anderson Medical Center	gift from Dr. Isaiah Fidler
Human: A375c28	M.D. Anderson Medical Center	gift from Dr. Isaiah Fidler
Human: WLH6215	The Wistar Institute	a gift from Dr. Meenhard Herlyn

**Experimental models: Organisms/strains**		

HGFtg	Noonan, et al.^34^	Noonan, et al.^34^
PTENfl/fl	Dankort, et al.^35^	Dankort, et al.^35^
Tyr:CreER tg	Dankort, et al.^35^	Dankort, et al.^35^

**Oligonucleotides**		

primers for Entpd5 promoter –2827 to –2627: forward: cttctggaagagagcaaatg reverse: gcagacaccaccgtgcccg	This paper	
primers for Entpd5 promoter –840 to –641: forward: tcattgtctcctcccattcc reverse: tcagtcagtcatgagcgcc	This paper	
primers for Entpd5 promoter –222 to –25: forward: ggcctagcctgttgtactgg reverse: gctttgttgctgaagcagtg	This paper	
primers for Entpd5 RT-PCR: forward: ggctggcctcaaactcatag reverse: acacacacagcatccaccat	This paper	
primers for Atf6 RT-PCR: forward: agtgtattacgcctcccctg reverse: agtcctgcccattgatcaca	This paper	
primers for Timp2 RT-PCR: forward: gtgacttcattgtgccctgg reverse: tctcttgatgcaggcgaaga	This paper	
primers for Pak3 RT-PCR: forward: tgcatggggatgtctgttta reverse: ctgatggcagcttctgtgtc	This paper	
primers for β-actin RT-PCR: forward: cctctatgccaacacagtgc reverse: cctgcttgctgatccacatc	This paper	

**Recombinant DNA**		

pGFP-PTEN	This paper	
pGFP-PTEN	This paper	
pGFP-PTEN	This paper	
pBabe-PTEN	Addgene	
pBabe-PTEN	addgene	
pBabe-PTEN	addgene	
pBabe-IGF1R	addgene	
ATF6 WT	addgene	
ATF6 M	addgene	
IGF1R shRNA (2)	Thermo scientific	RMM4431/RHS4430
Entpd5 shRNA (set)	Thermo scientific	RMM4532-EG12499
pMSCV antisense Entpd5	This paper	
pMSCV Entpd5	This paper	
PTEN shRNA	This paper	

**Software and algorithms**		

GraphPad Prism 6 software		
ImageScope V 10.0 software	Aperio Technologies	
Openlab software	Improvision/PerkinElmer	
Partek Genomics Suite software	Partek	

### Resource availability

#### Lead contact

Further information and requests for resources and reagents should be directed to and will be fulfilled by the lead contact, Yanlin Yu (yuy@mail.nih.gov).

#### Materials availability

Presented materials are made available upon reasonable request to the lead contact.

### Experimental model and subject details

#### Animal models

HGF^Tg^, PTEN^fl/fl^ and Tyr:CreER^Tg^ mice were described previously.^34^^,^^35^ HGF^Tg/+^/PTEN^fl/fl^/Tyr:CreER^Tg/+^ and HGF^Tg/+^/PTEN^fl/fl^/Tyr:CreER^+/+^ mouse cohorts were developed by crossing HGF^Tg/+^, PTEN^fl/fl^ and Tyr:CreER^Tg/Tg^ mice and genotyping as previously described.^34^^,^^35^ The depletion of PTEN was achieved by administration of 4-hydroxytamoxifen (4-OHT) and assessed by PCR.

#### Plasmids, antibodies, cell lines and culture

Plasmids: pGFP-PTEN WT, pGFP-PTEN G129E and pGFP-PTEN C124A, a generous gift from Dr. Kenneth M. Yamada, were subcloned into Tet-off (on) inducible expressing vector MG3.^37^ pBabe-PTEN WT, pBabe-PTEN G129E, pBabe-PTEN C124S, pBabe-IGF1R, ATF6 wildtype and mutant expressing vectors and reporter plasmid were provided by Addgene (Cambridge, MA). Human and mouse IGF1R and Entpd5 shRNAs expressing plasmids, Entpd5 cDNA and ATF6 siRNA were purchased from GE Dharmacon (Lafayette, CO). Entpd5 sense and anti-sense expressing plasmids were constructed in pMSCV vector. PTEN shRNA expressing plasmid was constructed in the pSUPER vector employing synthesized double stranded DNA fragments directed against nucleotides of the PTEN coding region (mouse BC021445, 655 - 675 and human BC005821, 1283-1303). Antibodies: anti-PTEN antibody was obtained from CASCADE biosciences (Winchester, MA), whileanti-phospho-AKT (Ser473), anti-phospho-AKT (Thr308), anti-AKT, anti-phospho-ERK 1/2 (Thr202/Tyr204), anti-ERK1/2, anti-IGF1Rβ, anti-GSK3α, anti-phospho-GSK3α(Ser21), anti-GSK3β, anti-phospho-GSK3β (Ser9), anti-p70S6, anti-phospho-p70S6 (Thr389), anti-4EBP1, anti-phospho-4EBP1 (Thr37/46) and anti-PTEN antibodies were obtained from Cell Signaling (Danvers, MA, USA). Anti-IGF1R antibody was obtained from Sigma-Aldrich (Saint Louis, MO), anti-Atf6 and anti-Entpd5 antibody from Abcam (Cambridge, MA), anti-EGFR antibody from Millipore (Billerica, MA), anti-Met and anti-β-actin antibodies from Santa Cruz (Dallas, TX) and anti-atf6 from Thermo Scientific (Rockford, IL, USA). PHA-E was purchased from US Biological (Swampscott, MA). B16F1 cell line was obtained from the AmericanType Culture Collection (ATCC, Manassas, VA). The 37-7 cells were derived from melanoma in HGF transgenic mice.^37^ Panel cell lines of A375p, A375sm, A375c5 and A375c28 were a generous gift from Dr. Isaiah Fidler (M.D. Anderson Medical Center, Houston, TX). WLH6215 cell line was a gift from Dr. Meenhard Herlyn (The Wistar Institute, Philadelphia, PA). The stable expressing cells were established through transfection using lipofectamine 2000 reagent (Invitrogen, Carlsbad, CA) and selected by antibiotics G418 or puromycin (Sigma, St. Louis, MO).

### Method details

#### Tissue microarray (TMA) analysis

Human ME207, ME208 and ME1004b tissue microarrays (TMA) contain nevus, primary melanomas and lymph metastatic melanomas (US Biomax, Rockville, MD). TMA slides were stained with anti-PTEN, anti-Endpd5 anti-IGF1R and ATF6 antibodies and substrate DAB or AEC or permanent red (DAKO Cytomation, Carpinteria, CA). TMA slides were scanned by ImageScope and reviewed by three pathologists. Immunoreactivity scores were analyzed by three pathologists using ImageScope V 10.0 software (Aperio Technologies, Vista, CA). Immunostaining intensities were scored strong as 3; moderate as 2; week as 1 and negative as 0. Frequency of labeled tumor cells was scored 100% as 5, 66-99% as 4, 33-65% as 3, 10 -32% as 2, <10% as 1, no cell as 0. IHC scores equal intensity plus frequency.

#### Western blot analysis

Immunoblots were performed on lysates generated from cultured cells and tissues solubilized in RIPA buffer.^66^

#### Relative quantitative reverse transcriptase polymerase chain reaction (qRTPCR)

qRTPCR were performed on total RNA generated from cells using RNAeasy kit (Qiagen).^66^^,^^67^

#### Motility and invasion assay

Cell motility and invasion were measured as described.^66^^,^^68^^,^^69^

#### Cell proliferation

^3^H-thymidine incorporation assay and CCK8 kit were used for the measurement of cell growth.^37^^,^^66^

#### Luciferase reporter assays

Luciferase assays were performed as described.^67^

#### Chromatin immunoprecipitation (ChIP)

ChIP was performed as described.^66^^,^^67^ Immunoprecipitated DNAs were analyzed by PCR using the following primers for Entpd5 promoter:–2827 to –2627, cttctggaagagagcaaatg and gcagacaccaccgtgcccg;–840 to –641, tcattg tctcctcccattcc and tcagtcagtcatgagcgcc;–222 to –25, ggcctagcctgttgtactgg and gctttgttgctgaagcagtg.

#### Experimental and spontaneous metastasis assays

Cells were intravenously injected via a tail vein or footpad into 4 – 6 week-old male mice. There were six different parent cell lines: B16F1 cells were injected at 5x10^5^ into FVB/BL6 or 1x10^5^ into athymic nude; 37-7 cells were injected at 2x10^5^ into FVB or 1x10^5^ into athymic nude; human melanoma A375 panel cells were injected via tail vein or footpad into NSG mice at 1x10.^6^ Tumor numbers were obtained by visual inspection of tissues in mice euthanized 21 days post-transplantation, and micrometastases were counted by pathologist’s evaluation after dissection of the lung.^37^^,^^66^^,^^68^^,^^69^ All mouse procedures were performed according to National Institutes of Health guidelines. The animal studies were under the animal proposal LMB042, approved by the National Cancer Institute-Bethesda Animal Care and Use Committee (ACUC) in the United States of America.

#### Immunohistochemistry

Lung tissues were fixed in 10% buffered formalin solution (pH 7.2) for 16 h, and/or frozen in OCT compound and serially sectioned to 15 μm at 20°C. Immunohistochemistry was performed as described.^69^ Immunoreactivity scores were analyzed using ImageScope V 10.0 software from Aperio Technologies (Vista, CA). The size of metastases was quantified by ImageJ software and analyzed using Statgraphics software.

#### Xenograft model

1x10^5^ B16F1 cells stably expressing PTEN WT, PTEN ΔL or PTEN ΔLP mutants, as well as an empty vector, were subcutaneously injected into athymic nude mice. Tumors were measured from day 7 after injection. The volumes were estimated using the formula: volume = width^2^ × length/2.^70^^,^^71^

#### *Ex vivo* microscopy

Cells were labeled with the Cell Tracker Green CMFDA (Invitrogen) for 30 minutes at a final concentration of 25 Amol/L in serum-free DMEM, and then normalized for 30 minutes in normalmedia. The Cell Tracker Green CMFDA-labeled cells were trypsinized, detached, and re-suspended in 1ml HBSS to a concentration of 10x10^6^ cells/ml. Using a 27G1/2 gauge needle, 0.1 ml cells (1x10^6^) were delivered to 4-6 weeksold female athymic nude mice by lateral tail vein injection. Mice were euthanized at 1, 6, or 24 h after injection. Five mice were injected per cell line per time point. When mice were euthanized, the lungs were dissected for e*x vivo* imaging with a fluorescent microscope (Leica DM IRB). Images of 10 representative regions of each lung were captured using Openlab software (Improvision/PerkinElmer). The number of Green CMFDA-labeled fluorescent cells per image was counted using the Openlab software automatic feature counter and histograms were constructed in Microsoft Excel. Statistical significance was determined using one-way ANOVA and a Turkey honest significant difference (HSD) test. Experiments were performed in duplicate.^72^

#### Microarray analysis

RNAs from cells with various PTEN mutant forms with triplicate biological replications of each microarray experiment were isolated using Trizol. Total RNA was further purified on an RNAeasy column (Qiagen) and the RNA quality was checked by an Agilent Bioanalyzer (Agilent Technologies, Palo Alto, CA, USA). Target labeling and hybridization to GeneChips were carried out in the NCI Frederick Microarray Core facility using the GeneChip Mouse 430_2 Array purchased from Affimetrixs. The microarray signals were normalized using the RMA algorithm. The significantly expressed genes were selected based on ANOVA analysis by Partek Genomics Suite software (Partek, St. Charles, MO, USA). The ANOVA gene lists by P value of 0.05 and absolute value of fold change of 1.5 were used in the gene ontology analysis by the commercial gene pathway analysis web tool (http://trials.genego.com/cgi/index.cgi).

#### Analyses of clinical outcome data

Clinical follow-up and gene expression datasets were obtained from publicly available datasets (GSE7929, GSE53118, GSE54467 and GSE22138) in Gene Expression Omnibus (GEO).

### Quantification and statistical analysis

Statistical analyses were performed as follows: unpaired t-test (two-tailed) for all column datasets or Pearson r (two-tailed) analysis for correlation or Log-rank (Mantel-Cox) test for Kaplan Meier survival analysis using GraphPad Prism 6 software. The p values of less than 0.05 were considered statistically significant.

## Data Availability

• Data: TCGA data used for correlation analysis is publicly available from https://www.cbioportal.org/. Clinical follow-up and gene expression datasets were obtained from publicly available datasets (GSE7929, GSE53118, GSE54467 and GSE22138) in Gene Expression Omnibus (GEO). • Code: The microarray data have been deposited at a public functional genomics data repository in The National Center for Biotechnology Information (GSE210359) and are publicly available as of the date of publication. The accession number is listed in the key resources table. Any additional information required to reanalyze the data reported in this paper is available from the lead contact upon request.
