# Supplementary material for: PTEN phosphatase inhibits metastasis by negatively regulating the Entpd5/IGF1R pathway through ATF6
Source: iScience. 2023 Jan 26;26(2):106070. doi: 10.1016/j.isci.2023.106070 (PMC9942123; doi:10.1016/j.isci.2023.106070)

iScience, Volume *26*

Supplemental information

PTEN phosphatase inhibits metastasis by negatively regulating the Entpd5/IGF1R pathway through ATF6

Yanlin Yu, Meng Dai, Liping Huang, Weiping Chen, Ellen Yu, Arnulfo Mendoza, Helen Michael, Chand Khanna, Marcus Bosenberg, Martin McMahon, and Glenn Merlino

Supplemental information

Fig. S1. PTEN downregulation correlates with worse survival in SKCM-TCGA. Related to Figure 1. The patients with PTEN downregulation (red, n=53) had significantly worse survival than the patients with PTEN unalteration (blue, n=317) in SKCM-TCGA (Log-rank test p-value: 0.0456).

Fig. S2. **The correlation of PTEN mutations and human metastatic melanoma. Related to Figure 1.** The metastatic melanoma patients with PTEN mutations (deletion and mutation, red, n=18) have significantly worse survival than the patients without PTEN mutations (blue, n=48) in SKCM-TCGA metastatic melanoma (MSKCC, JCO Precis Oncol 2017) data set (Log-rank test p-value: 0.00935).

Fig. S3. LinkedOmics (Vasaikar SV et al. Nucleic Acids Res. 2018, 46:D956-D963) analysis of genes methylation. Related to Figure 1. Analysis shows that PTEN methylation is a negative correlation with survival probability in SKCM-TCGA data (A); in contrast, methylation of ATF6 (C) and ENDPT5 (B) is a positive correlation with survival probability in SKCM-TCGA.

Fig. S4. Representative H&E stained lung sections for the experimental metastasis. Related to Figure 1. (A) Relative lung metastases are indicated in the panel for A375p transfected with shRNA for PTEN. C, empty vector; #6, #9 and #18, knockdown clones. (B) Relative lung metastases are indicated in the panel for A375sm transfected with PTEN. C, empty vector; PTEN, overexpression of PTEN. (C) Relative lung metastases are indicated in the panel for A375c28 transfected with PTEN. C, empty vector; PTEN, overexpression of PTEN. (D) Relative lung metastases are indicated in the panel for B16F1 transfected with PTEN. C, empty vector; WT, overexpression of PTEN. (E)

Relative lung metastases are indicated in the panel for B16F1 transfected with shRNA for PTEN. C, empty vector; shRNA, knockdown of PTEN. Scale bar = 5 mM.

Fig. S5. Representative lung metastases from the experiment sectioned and stained with hematoxylin and eosin (H&E). Related to Figures 2 and 3. (A) and (B) Lung images from host FVBxBL6 (A) and nude (B) mice are indicated in the panel for B16F1 transfected with empty vector (C) PTEN wildtype (WT) or PTEN phosphatase mutant (ΔLP). (C and D) Lung images from host FVB (C) and nude (D) mice are indicated in the panel for 37-7 cells transfected with empty vector (C) PTEN wildtype (WT) or PTEN phosphatase mutant (ΔLP). Scale bar = 5 mM. (E) Quantitative data of metastatic tumor size (area) from lung sections with metastases from mice bearing B16F1 cells with empty vector (C), PTEN WT (WT), PTEN lipid phosphatase deficient (ΔL), PTEN phosphatases dead mutant (ΔLP). Compared to control or other PTEN groups, the size of metastatic tumors in PTEN lipid phosphatase deficient (ΔL) significantly was increased (* f-test, p=0.0000; t-test, p=0.024). (F) PTEN WT (WT) could significantly inhibit tumor growth in xenografts in nude mice (p=0.001). Both PTEN lipid phosphatase deficient (ΔL) and PTEN phosphatase dead mutant (ΔLP) increased tumor growth slightly. (G, H and I) B16 cells stably expressing PTEN wt (WT), PTENΔL(ΔL) and PTENΔLP (ΔLP) mutants, as well as empty vector (C), were subcutaneously injected into athymic nude mice. After the tumors have grown to an 8 mm^3 size, the tumors were sectioned from euthanized mice and fixed and dissected for evaluating the volume of BrdU (proliferation) (G), Tunnel (apoptosis) (H) and CD31 (microvessel) (I) by immunohistochemistry staining. Data is representative of three independent experiments.

Graphs show the mean ±SEM. The p-value is shown by an unpaired t-test (two-tailed). (J) A summary of biological behaviors for PTENs.

Fig. S6. PTEN mutation in all TCGA cancer data set (n=1321). Related to Figure 2. 12.6% mutation of PTEN happened in the R130 position, which lost the phosphatase activity, implying that protein phosphatase is important in tumorigenesis.

Fig. S7. PTEN wildtype and ∆L mutant inhibited the metastasis whereas ∆LP mutant enhanced the metastasis, a protein phosphatase deficient mutant (∆P) also promoted the metastasis in B16F1 (A and B) and RMS772 (C and D) cells. Related to Figure 2. Overexpression of PTEN wildtype (WT), PTEN lipid phosphatase deficient (ΔL), phosphatase dead mutant (ΔLP) and protein phosphatase deficient (ΔP) in B16F1 (A and B) and RMS772 (C and D) cells. The Elisa assay determined the level of PTEN protein in B16F1 (A) and RMS772 (C). (B) and (D) The number of gross lung metastasis in B16F1 transfectants (B) and RMS772 transfectants (D). Data represented as mean ±SEM for all columns. The p-value is shown by an unpaired t-test (two-tailed).

Fig. S8. The gene sets are associated with transcript changes by PTEN wildtype (WT) (A), lipid phosphatase mutant (∆L) (B) or phosphatase dead mutant (∆LP) (C) in B16F1 cells compared with empty vector (control). Related to Figure 4. The significantly expressed genes were filtered by a P value of 0.05 and an absolute value of fold change of 1.5 in ANOVA analysis using Partek Genomics Suite software and gene ontology analysis.

Fig. S9. Representative H&E stained lung sections for the experimental metastasis. Related Figures 5 and 6. (A) Relative lung metastases are indicated in the panel for A375sm transfected with antisense for human Entpd5. C, empty vector; As, antisense

plasmids. (B) Relative lung metastases are indicated in the panel for B16F1-PTEN transfected with Entpd5. C, empty vector; Entpd5, overexpression of Entpd5. (C) Relative lung metastases are indicated in the panel for A375p transfected with Entpd5. C, empty vector; Entpd5, overexpression of Entpd5. (D) Relative liver metastases are indicated in the panel for A375p transfected with Entpd5. C, empty vector; Entpd5, overexpression of Entpd5. (E) Relative lung metastases are indicated in the panel for B16F1-PTENΔLP transfected with shRNA for IGF1R. C, empty vector; sh1 and sh2, clones for knockdown of IGF1R. (F) Relative lung metastases are indicated in the panel for A375sm transfected with shRNA for IGF1R. C, empty vector; sh1 and sh2, clones for knockdown of IGF1R. (G) Relative lung metastases are indicated in the panel for B16F1-PTEN transfected with IGF1R. C, empty vector; IGF1R, overexpression of IGF1R. Scale bar = 5 mM.

Fig. S10. Immunohistochemistry analyses of tissue microarrays were performed using antibodies against PTEN, Entpd5, IGF1R and ATF6 in consecutively cut sections of nevi (n=18), melanomas (n=56) and metastatic melanomas (n=26). Related to Figure 7. Pearson R correlation analysis of PTEN and Entpd5 (A), PTEN and IGF1R (B), PTEN and ATF6 (C) or Entpd5 and IGF1R (D), Entpd5 and ATF6 (E) and IGF1R and ATF6 (F) expression using tumor staining scores [average intensity of labeled tumor cells (none =0, low = 1, medium = 2 and high = 3) plus the frequency of tumor labeling (no cells = 0, <10% = 1, 10-32% = 2, 33-65%= 3, 66-99% = 4 and 100% = 5).

Fig. S11. In the study of the GSE53118 dataset with late-stage malignant melanomas (n=79). Related to Figure 7. The correlation of PTEN with Entpd5 (A), PTEN with IGF1R (B) and Entpd5 with IGF1R (C) was performed by two-tailed Pearson r analysis,

and Kaplan Meier survival curves (D, E) were analyzed by Log-rank (mantel-Cox) test using GraphPad Prism 6 software.

Fig. S12. In the study of the GSE54467 dataset with late-stage malignant melanomas (n=79). Related to Figure 7. The correlation of PTEN with Entpd5 (A), PTEN with IGF1R (B) and Entpd5 with IGF1R (C) was performed by two-tailed Pearson r analysis, and Kaplan Meier survival curves (D, E) were analyzed by Log-rank (mantel-Cox) test using GraphPad Prism 6 software.

Fig. S13. In the study of the GSE22138 dataset with malignant melanomas (n=63). Related to Figure 7. The correlations of PTEN with Entpd5 (A) and PTEN with IGF1R (B) were performed by two-tailed Pearson r analysis using GraphPad Prism 6 software.

Fig. S14. Entpd5 expression correlates with ATF6 (r=0.42) in SKCM-TCGA data. Related to Figure 8.

Fig. S1

PTEN downregulation correlates with worse survival in SKCM-TCGA.

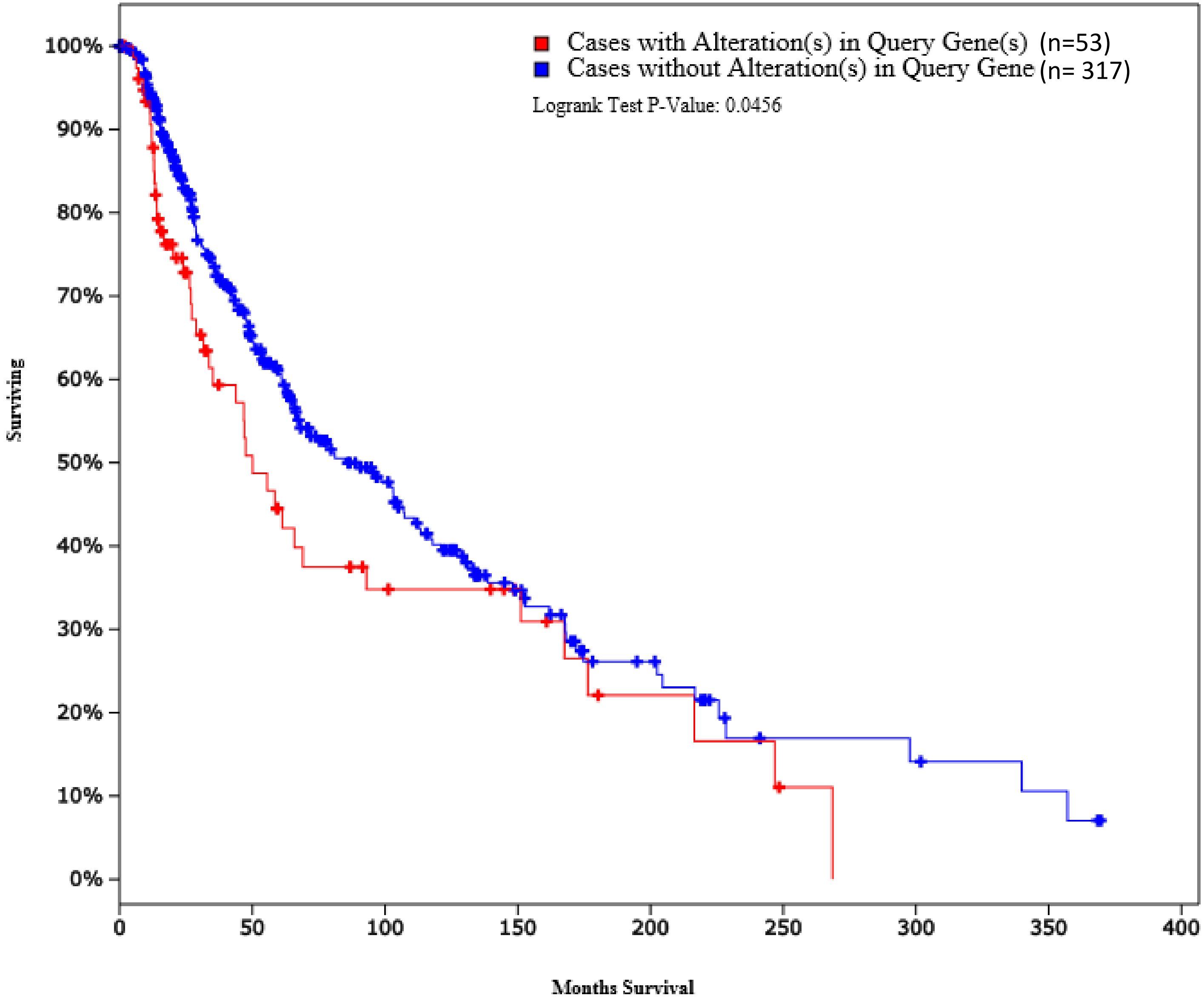

Fig. S2

The metastatic melanoma patients with PTEN mutations (deletion and mutation) have significantly worse survival in SKCM-TCGA metastatic melanoma (MSKCC, JCO Precis Oncol 2017) data set.

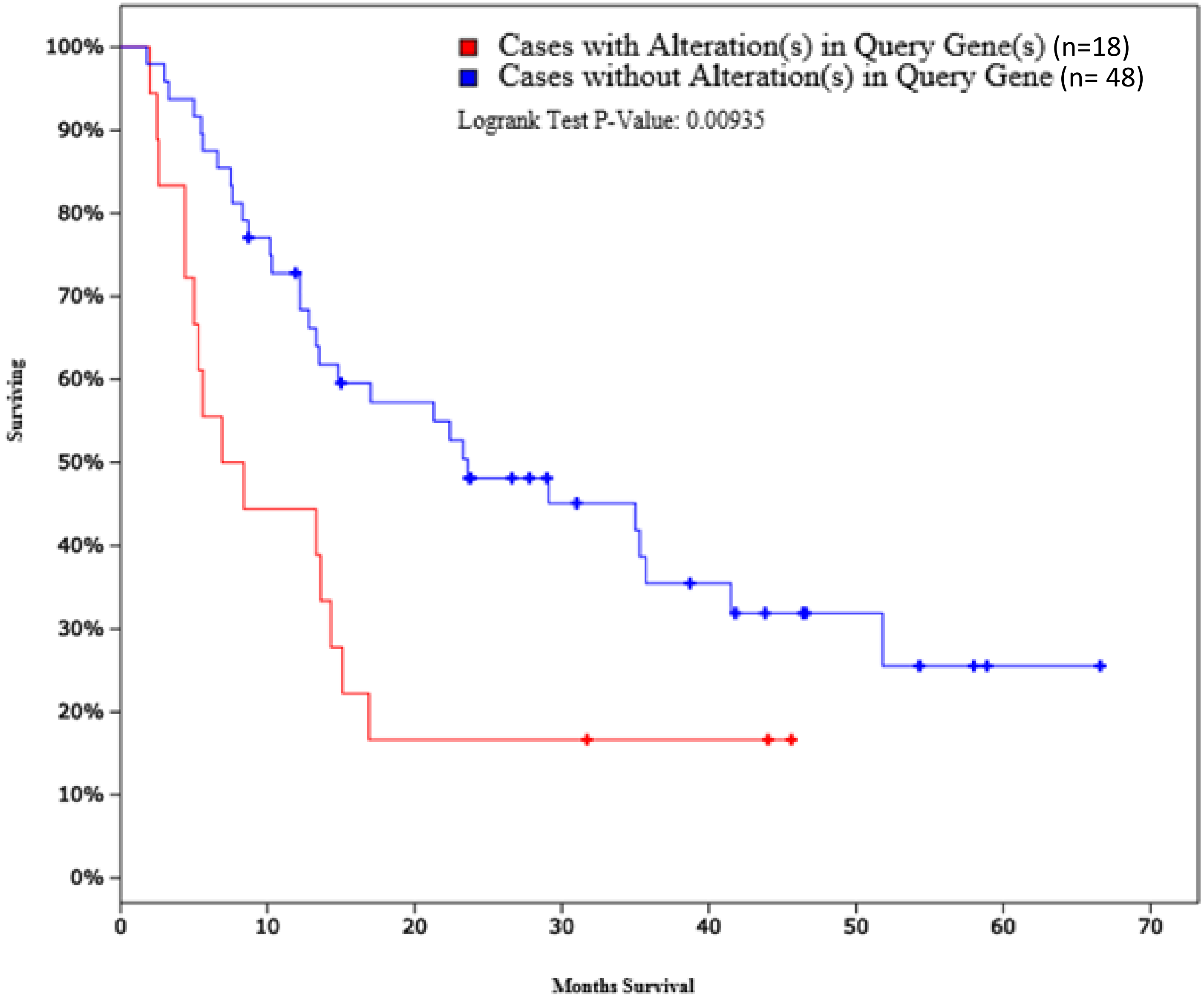

Fig. S3

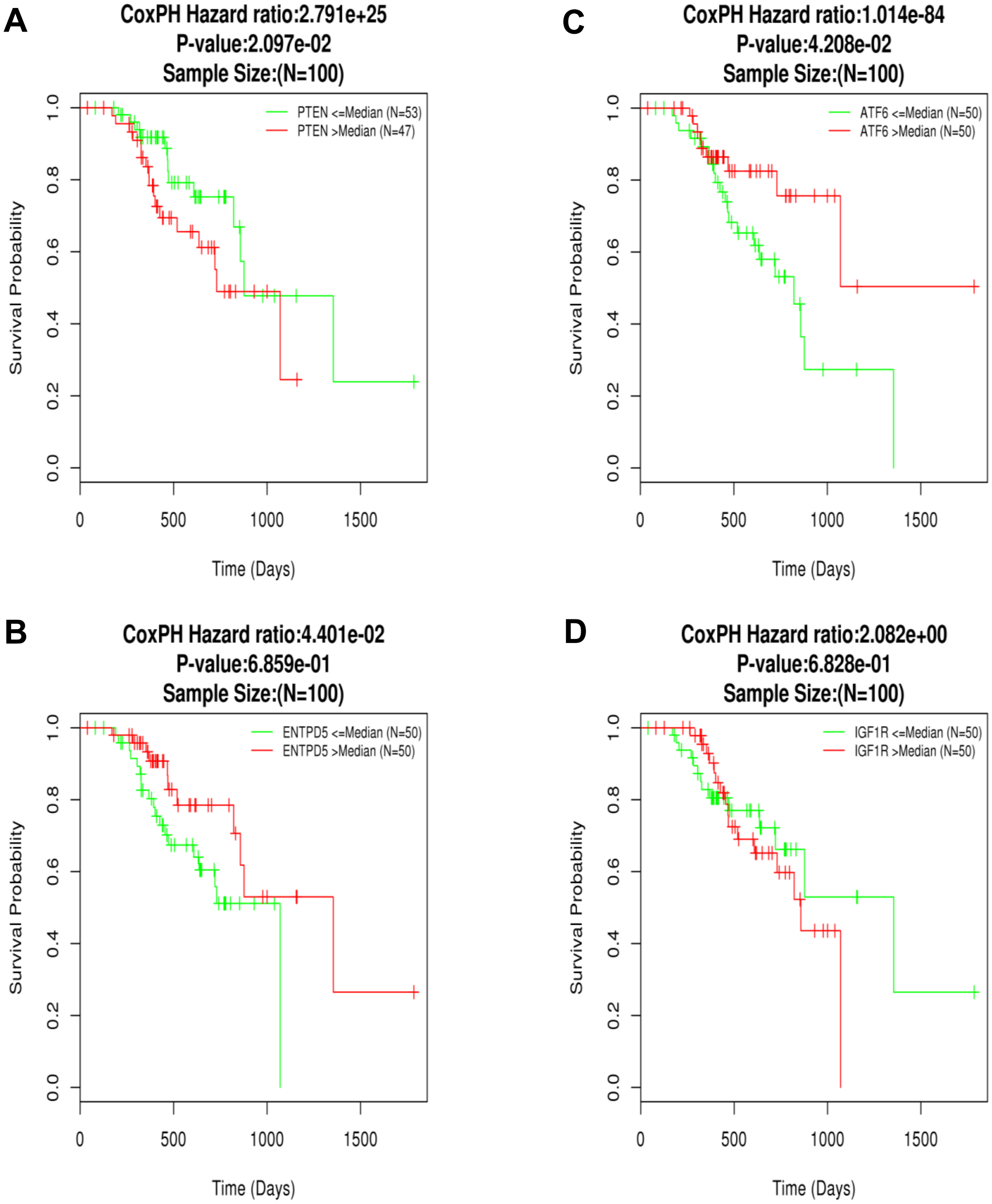

A
CoxPH Hazard ratio:2.791e+25
P-value:2.097e-02
Sample Size:(N=100)
PTEN <=Median (N=53)
PTEN >Median (N=47)
C
CoxPH Hazard ratio:1.014e-84
P-value:4.208e-02
Sample Size:(N=100)
ATF6 <=Median (N=50)
ATF6 >Median (N=50)
B
CoxPH Hazard ratio:4.401e-02
P-value:6.859e-01
Sample Size:(N=100)
ENTPD5 <=Median (N=50)
ENTPD5 >Median (N=50)
D
CoxPH Hazard ratio:2.082e+00
P-value:6.828e-01
Sample Size:(N=100)
IGF1R <=Median (N=50)
IGF1R >Median (N=50)
Survival Probability
0.0
0.2
0.4
0.6
0.8
1.0
Time (Days)
0
500
1000
1500

Fig. S4

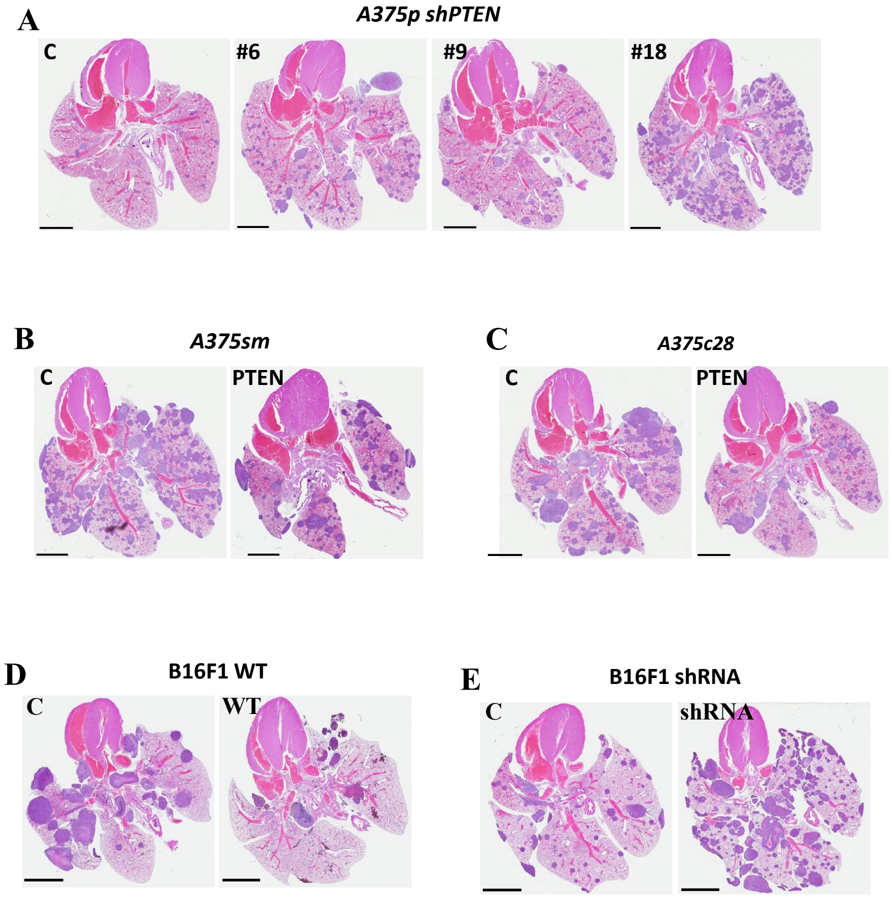
A
A375p shPTEN
C
#6
#9
#18
B
A375sm
C
PTEN
C
A375c28
C
PTEN
D
B16F1 WT
C
WT
E
B16F1 shRNA
C
shRNA

Fig. S5

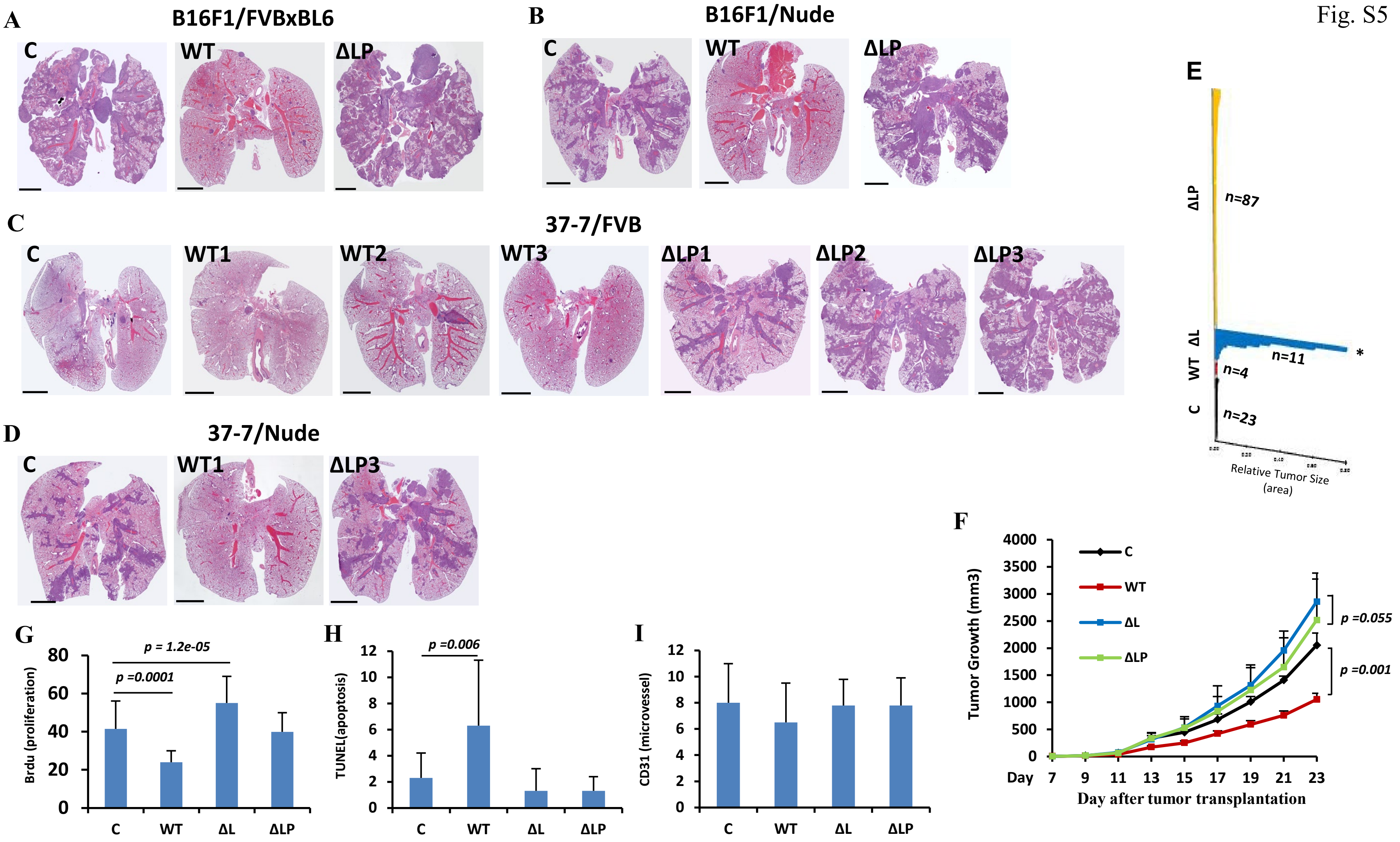

J **Biological behaviors of PTENs**

PTEN	mutation	In Vitro: 3H thymidine	In Vitro: motility	In Vitro: invasion	In vivo: SQ	In vivo: Brdu	In vivo: apoptosis	In vivo: CD31	In vivo: survival	In vivo: mets	In vivo: mets size
WT	non	–	–	–	–	–	†	no	–	–	no
ΔL	G129E	†	no	no	†	†	no	no	–	no	†
ΔLP	C124A	†	no	†	±	no	no	no	†	†	no

-, inhibition; +, stimulation or promotion; no, no effect. mets, metastasis; mets size, size of metastasis.

PTEN Mutation Position in TCGA data

(n= 1321)

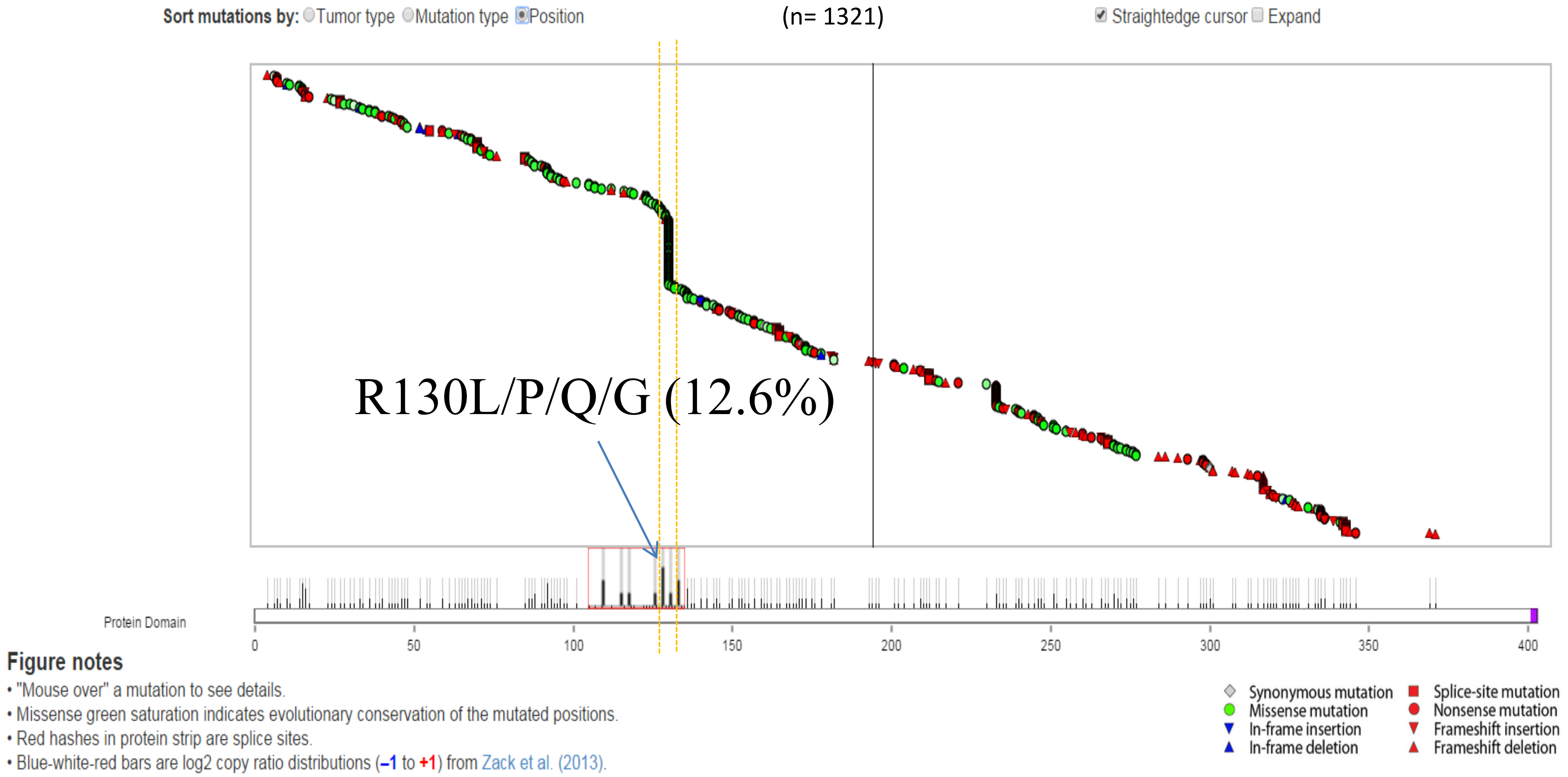

mutation site	function loss	% of total samples
G129	lipid	0.9
C124	protein & lipid	0.4
R130	protein & lipid	12.6

Fig. S7

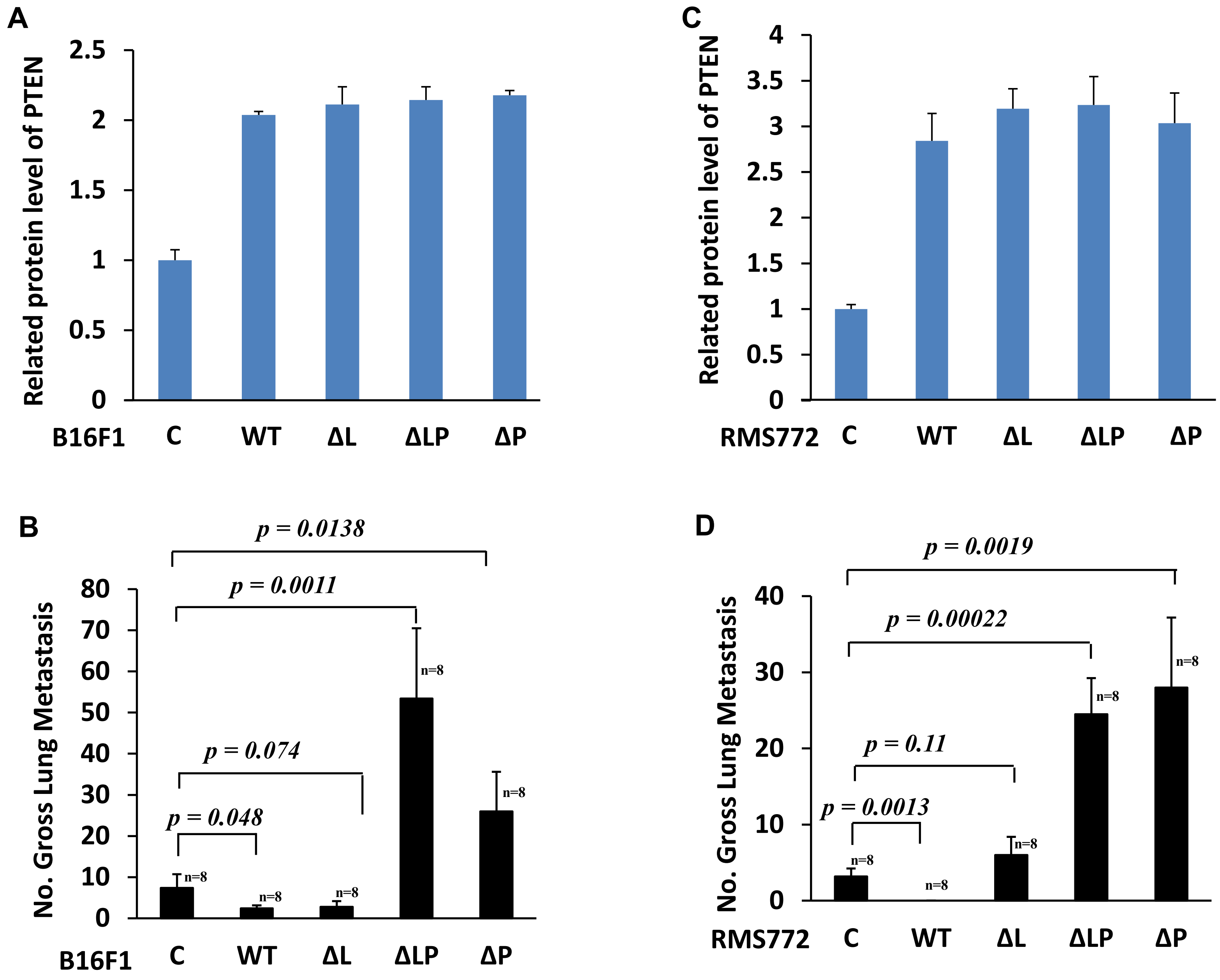
A
Related protein level of PTEN
2.5
2
1.5
1
0.5
0
B16F1
C
WT
ΔL
ΔLP
ΔP
C
Related protein level of PTEN
4
3.5
3
2.5
2
1.5
1
0.5
0
RMS772
C
WT
ΔL
ΔLP
ΔP
B
p = 0.0138
p = 0.0011
p = 0.074
p = 0.048
No. Gross Lung Metastasis
80
70
60
50
40
30
20
10
0
n=8
n=8
n=8
n=8
n=8
B16F1
C
WT
ΔL
ΔLP
ΔP
D
p = 0.0019
p = 0.00022
p = 0.11
p = 0.0013
No. Gross Lung Metastasis
40
30
20
10
0
n=8
n=8
n=8
n=8
n=8
RMS772
C
WT
ΔL
ΔLP
ΔP

Fig. S8

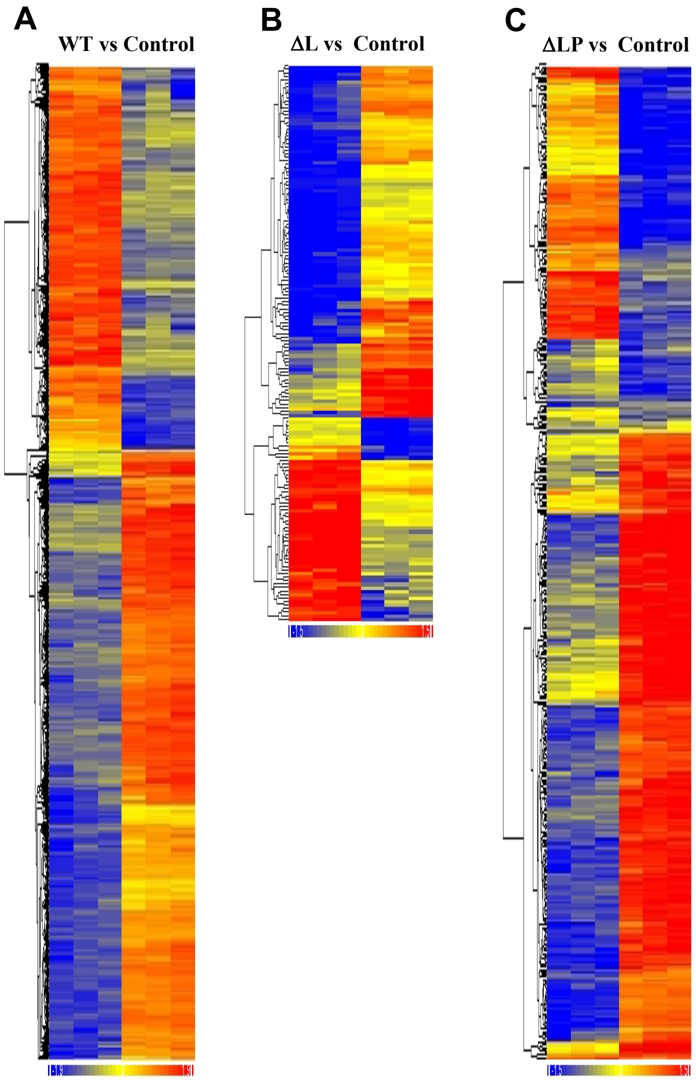

Fig. S9

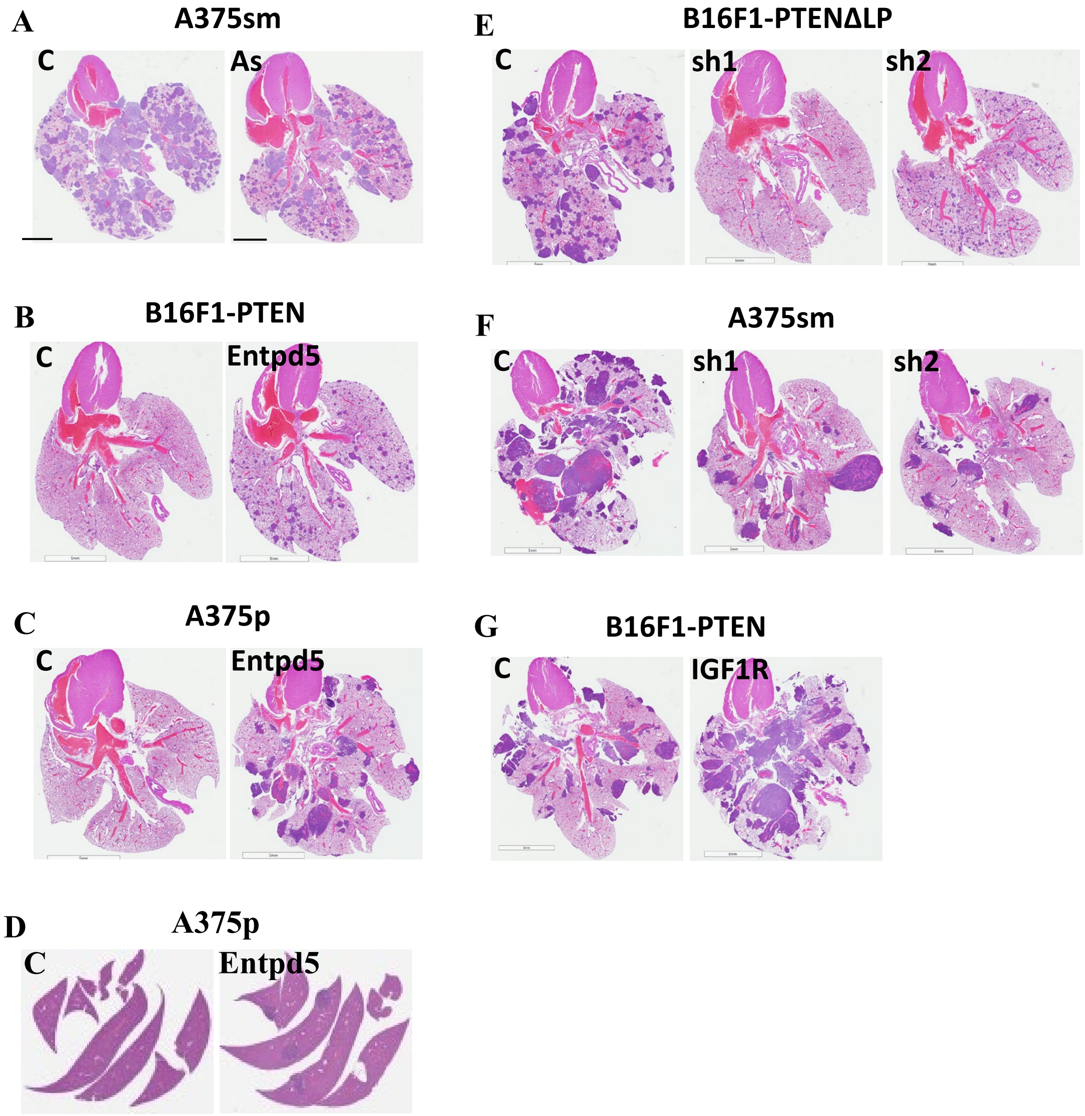

A
A375sm
C
As
B
B16F1-PTEN
C
Entpd5
C
A375p
C
Entpd5
D
A375p
C
Entpd5
E
B16F1-PTENΔLP
C
sh1
sh2
F
A375sm
C
sh1
sh2
G
B16F1-PTEN
C
IGF1R

Fig. S10

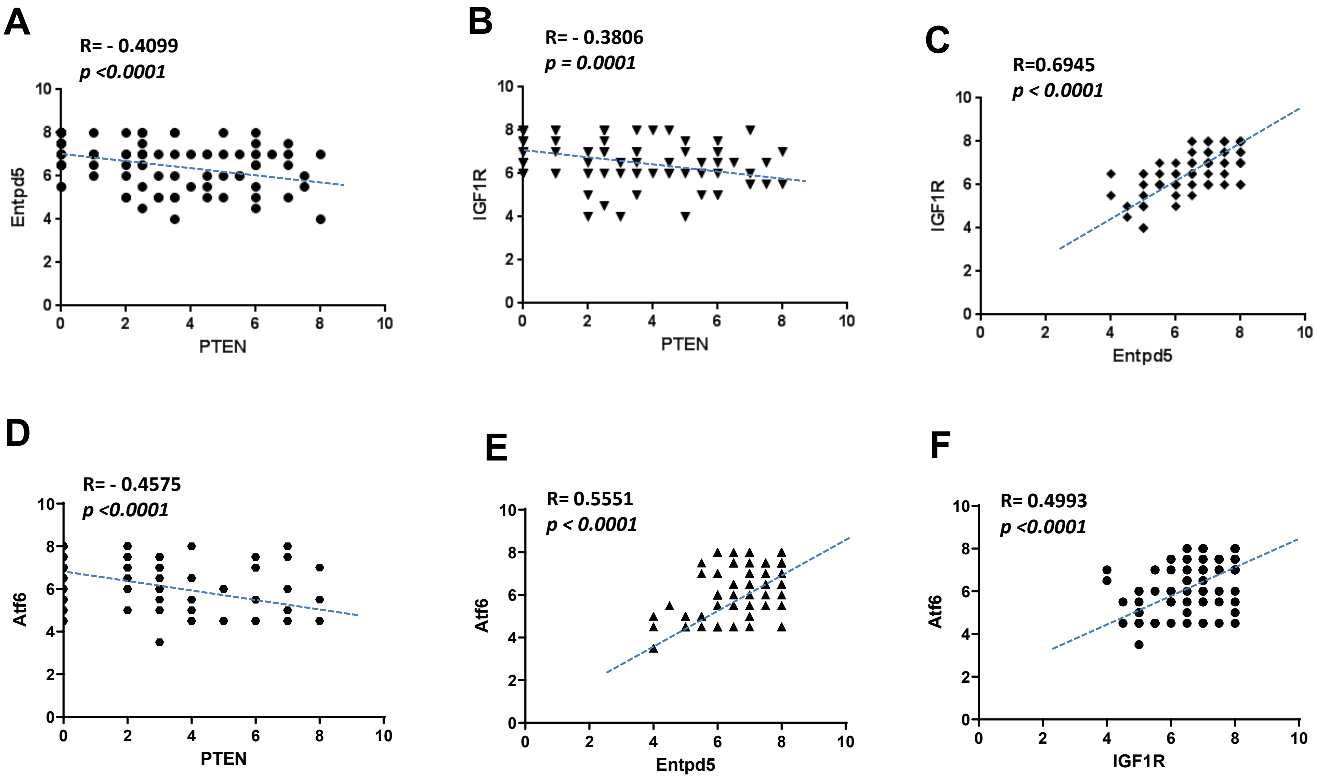
A
R= - 0.4099
p <0.0001
Entpd5
PTEN
B
R= - 0.3806
p = 0.0001
IGF1R
PTEN
C
R=0.6945
p < 0.0001
IGF1R
Entpd5
D
R= - 0.4575
p <0.0001
Atf6
PTEN
E
R= 0.5551
p < 0.0001
Atf6
Entpd5
F
R= 0.4993
p <0.0001
Atf6
IGF1R

Fig. S11

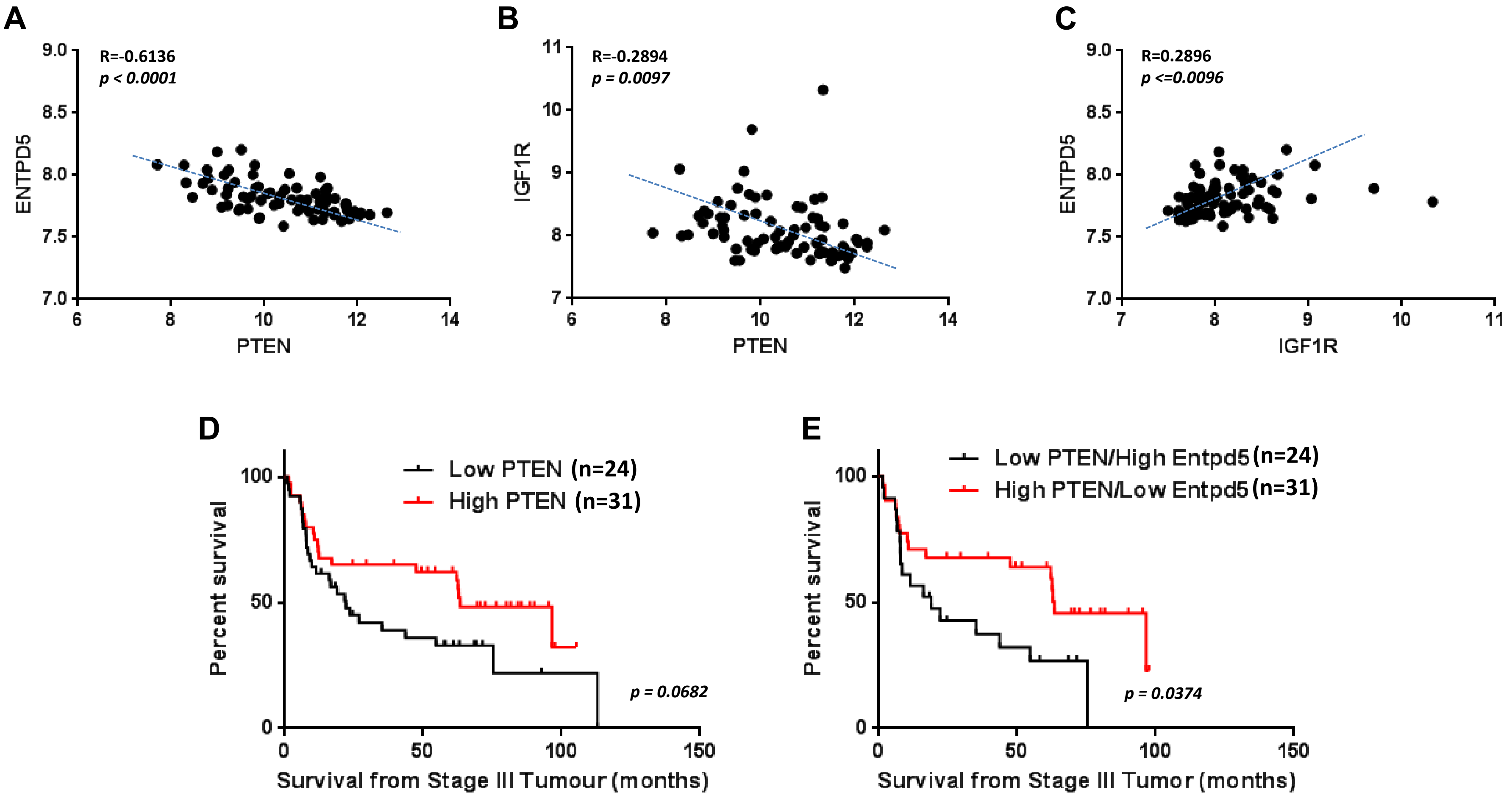
A
R=-0.6136
p < 0.0001
ENTPD5
PTEN
B
R=-0.2894
p = 0.0097
IGF1R
PTEN
C
R=0.2896
p <=0.0096
ENTPD5
IGF1R
D
Low PTEN (n=24)
High PTEN (n=31)
Percent survival
p = 0.0682
Survival from Stage III Tumour (months)
E
Low PTEN/High Entpd5 (n=24)
High PTEN/Low Entpd5 (n=31)
Percent survival
p = 0.0374
Survival from Stage III Tumor (months)

Fig. S12

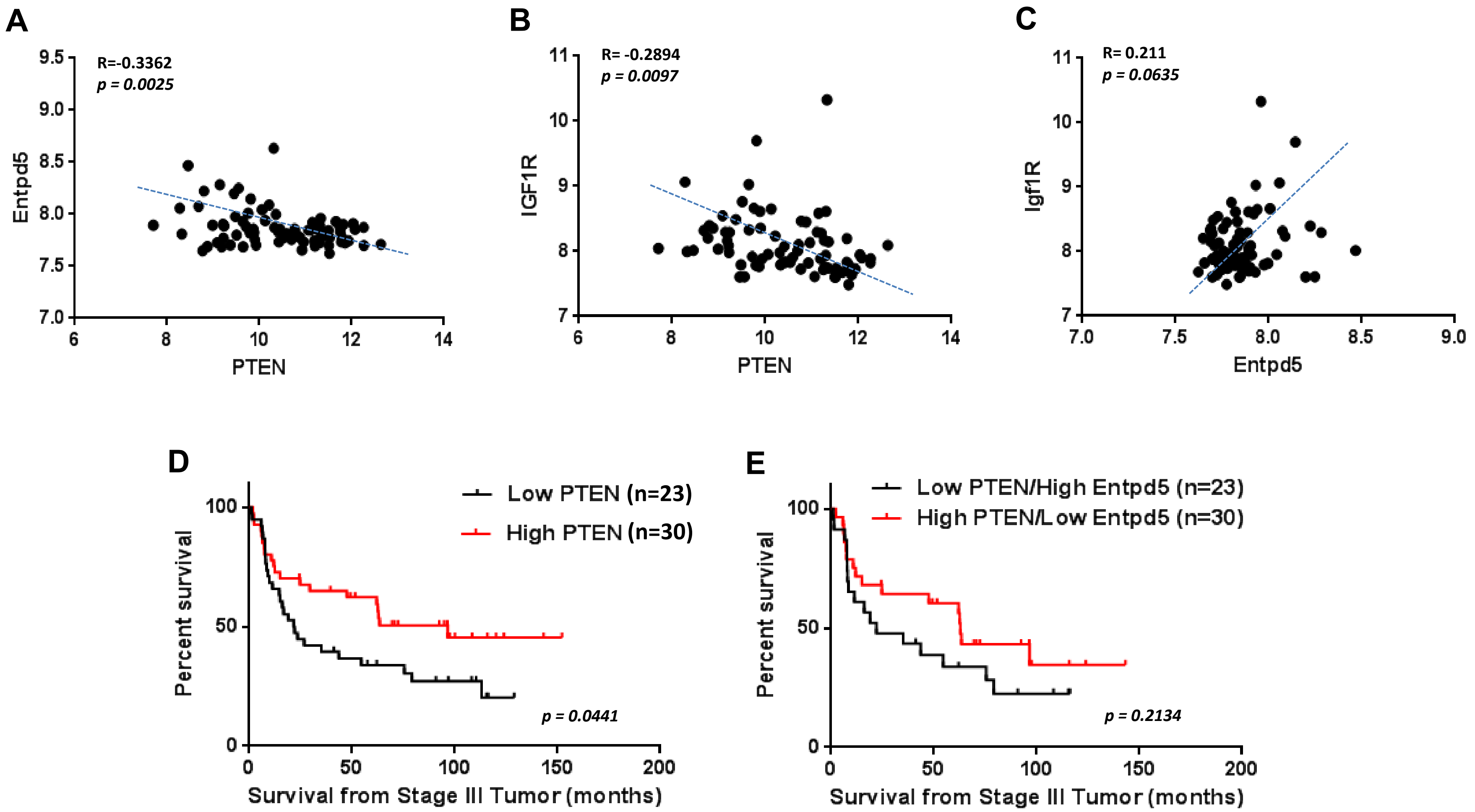
A
R=-0.3362
p = 0.0025
Entpd5
PTEN
9.5
9.0
8.5
8.0
7.5
7.0
6
8
10
12
14
B
R= -0.2894
p = 0.0097
IGF1R
PTEN
11
10
9
8
7
C
R= 0.211
p = 0.0635
Igf1R
Entpd5
7.0
7.5
8.0
8.5
9.0
D
Low PTEN (n=23)
High PTEN (n=30)
Percent survival
p = 0.0441
Survival from Stage III Tumor (months)
0
50
100
150
200
E
Low PTEN/High Entpd5 (n=23)
High PTEN/Low Entpd5 (n=30)
Percent survival
p = 0.2134
Survival from Stage III Tumor (months)

Fig. S13

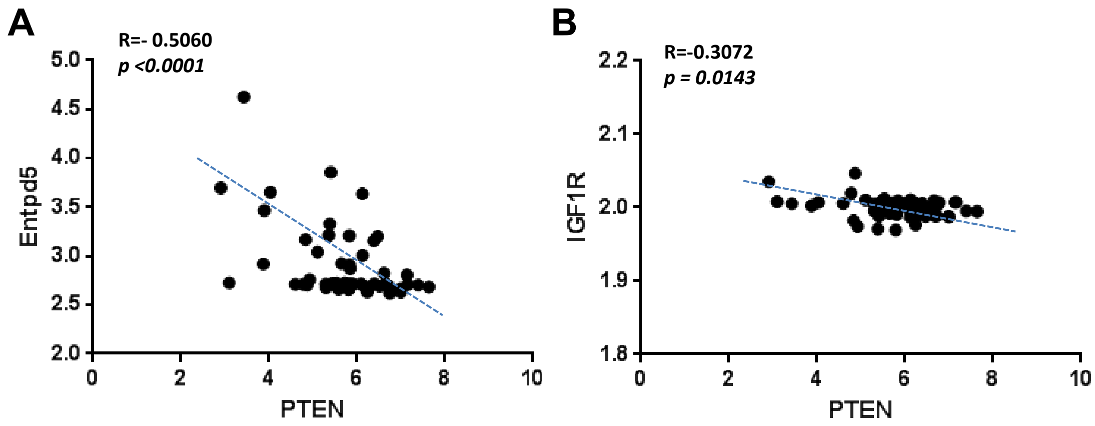

A
R=- 0.5060
p <0.0001
Entpd5
5.0
4.5
4.0
3.5
3.0
2.5
2.0
0
2
4
6
8
10
PTEN
B
R=-0.3072
p = 0.0143
IGF1R
2.2
2.1
2.0
1.9
1.8
0
2
4
6
8
10
PTEN

Fig. S14

Entpd5 expression correlated with ATF6 (r=0.42) in SKCM-TCGA data.

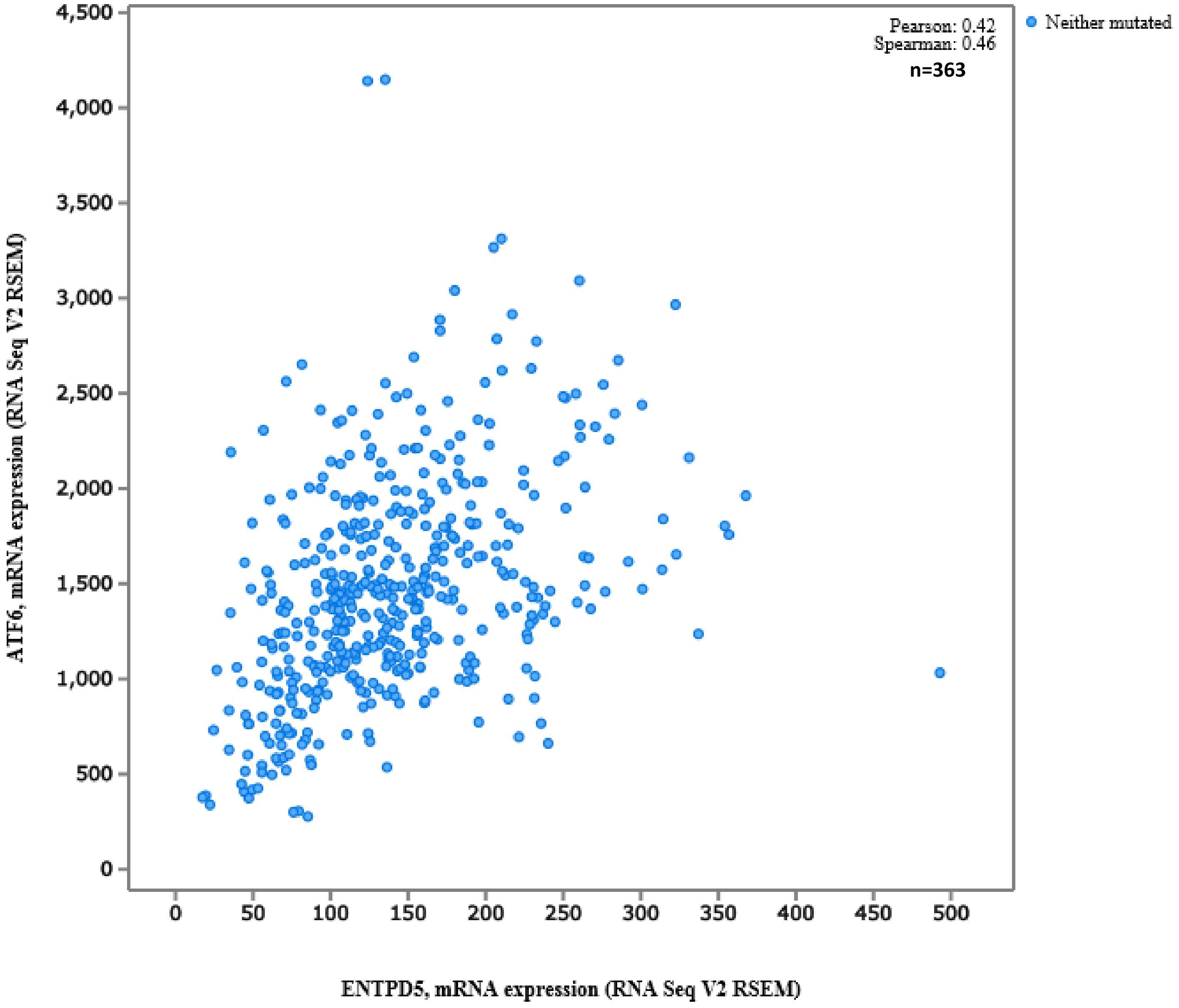

Supplement: Document S1. Figures S1–S14 [file mmc1.pdf]
